# Distinguishing Noise from Chaos: Objective versus Subjective Criteria Using Horizontal Visibility Graph

**DOI:** 10.1371/journal.pone.0108004

**Published:** 2014-09-23

**Authors:** Martín Gómez Ravetti, Laura C. Carpi, Bruna Amin Gonçalves, Alejandro C. Frery, Osvaldo A. Rosso

**Affiliations:** 1 Departamento de Engenharia de Produção, Universidade Federal de Minas Gerais, Belo Horizonte, Minas Gerais, Brazil; 2 Laboratório de Computação Científica e Análise Numérica (LaCCAN), Universidade Federal de Alagoas, Maceió, Alagoas, Brazil; 3 Instituto de Física, Universidade Federal de Alagoas, Maceió, Alagoas – Brazil; 4 Instituto Tecnológico de Buenos Aires (ITBA), Ciudad Autónoma de Buenos Aires, Argentina; 5 Departament de Física Fonamental, Universitat de Barcelona, Barcelona, Spain; 6 Instituto Politécnico. Centro Universitário UNA, Belo Horizonte, Minas Gerais, Brazil; Wake Forest School of Medicine, United States of America

## Abstract

A recently proposed methodology called the Horizontal Visibility Graph (HVG) [Luque *et al*., Phys. Rev. E., 80, 046103 (2009)] that constitutes a geometrical simplification of the well known Visibility Graph algorithm [Lacasa *et al*., Proc. Natl. Sci. U.S.A. 105, 4972 (2008)], has been used to study the distinction between deterministic and stochastic components in time series [L. Lacasa and R. Toral, Phys. Rev. E., 82, 036120 (2010)]. Specifically, the authors propose that the node degree distribution of these processes follows an exponential functional of the form 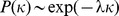, in which 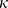 is the node degree and 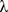 is a positive parameter able to distinguish between deterministic (chaotic) and stochastic (uncorrelated and correlated) dynamics. In this work, we investigate the characteristics of the node degree distributions constructed by using HVG, for time series corresponding to 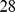 chaotic maps, 2 chaotic flows and 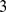 different stochastic processes. We thoroughly study the methodology proposed by Lacasa and Toral finding several cases for which their hypothesis is not valid. We propose a methodology that uses the HVG together with Information Theory quantifiers. An extensive and careful analysis of the node degree distributions obtained by applying HVG allow us to conclude that the Fisher-Shannon information plane is a remarkable tool able to graphically represent the different nature, deterministic or stochastic, of the systems under study.

## Introduction

Time series, temporal sequences of measurements or observations, are one of the basic tools for investigating natural phenomena. From time series analysis, one should judiciously extract information about the dynamics of the underlying process. Time series arising from chaotic systems share with those generated by stochastic processes several properties that make them very similar. Examples of these properties are: a wide-band power spectrum (PS), a delta-like autocorrelation function, and an irregular behavior of the measured signals. As irregular and apparently unpredictable behavior is often observed in natural time series, the question that immediately emerges is whether the system is chaotic (low-dimensional deterministic) or stochastic. If one is able to show that the system is dominated by low-dimensional deterministic chaos, then only few (nonlinear and collective) modes are required to describe the pertinent dynamics. If not, then, the complex behavior could be modeled by a system dominated by a very large number of excited modes which are in general better described by stochastic or statistical approaches.

The main objective of nonlinear time series analysis is the understanding of the dynamics of stochastic and chaotic processes. In recent years, a new few methods have been proposed to transform a single time series into a complex network, so that the dynamics of the process can be understood by investigating the topological properties of the network [1]–[6]. Essentially, this is a transformation from the time domain to the network domain, which allows for the dynamics of the time series to be studied via the organization of the network [4]. It was found that time series with different dynamics exhibit distinct topological structures. Specifically, noisy periodic signals correspond to random networks, and chaotic time series generate networks that exhibit small world and scale free features [1]. In brief, taking into account the results of this research line, one could say that the current literature suggests that network analysis can be used to distinguish different dynamic regimes in time series and, perhaps more importantly, that time series analysis can provide a powerful set of tools that augment the traditional network analysis toolkit to quantify networks in new and useful ways [5].

The distinction between stochastic and chaotic processes has received much attention, becoming one of the most appealing problems in time series analysis. Since stochastic and chaotic (low dimensional deterministic) processes share several characteristics, the discrimination between them is a challenging task. Time series with complex structures are very frequent in both natural and artificial systems. The interest behind this distinction relies in uncovering the cause of unpredictability governing these systems.

Much effort has being dedicated in the understanding of this topic. It was thought, in the origins of chaotic dynamics, that obtaining finite, non-integer values for fractal dimension was a strong evidence of the presence of deterministic chaos, as stochastic processes were thought to have an infinite value. Osborne and Provenzale [7] observed for a stochastic process a non-convergence in the correlation dimension (as a estimation of fractal dimension). They showed that time series generated by inverting power law spectra and random phases are random fractal paths with finite Hausdorff dimension and, consequently, with finite correlation dimension [7].

Among other methodologies to distinguish chaotic from stochastic time series we can mention the work of Sugihara and May [8] based on nonlinear forecasting in which they compare predicted and actual trajectories and make tentative distinctions between dynamical chaos and measurement errors. The accuracy of nonlinear forecast diminishes for increasing prediction time-intervals for a chaotic time series. This dependency is not found for uncorrelated noises [8]. Kaplan and Glass [9], [ 10] observed that the tangent to the trajectory generated by a deterministic system is a function of the position in phase space, consequently, all the tangents to a trajectory in a given phase space region will display similar orientation. As stochastic dynamics do not exhibit this behavior, Kaplan and Glass proposed a test based on these observations. Kantz and co-workers [11], [ 12] recently analyzed the behavior of entropy quantifiers as a function of the coarse-graining resolution, and applied their ideas to distinguish between chaos and noise. Their methodology can be considered a generalization of the Grassberger and Procaccia method [13] regarding the estimation of the correlation dimension and the consideration of finite values as signatures of deterministic behavior.

Chaotic systems display sensitivity to initial conditions which manifests instability everywhere in the phase space and leads to non-periodic motion (chaotic time series). They display long-term unpredictability despite the deterministic character of the temporal trajectory. In a system undergoing chaotic motion, two neighboring points in the phase space move away exponentially rapidly. Let 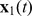 and 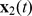 be two such points, located within a ball of radius 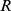 at time 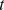. Further, assume that these two points cannot be resolved within the ball due to poor instrumental resolution. At some later time 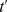 the distance between the points will typically grow to 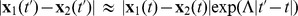, with 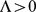 for a chaotic dynamics, being 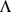 the biggest Lyapunov exponent. When this distance at time 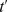 exceeds 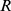, the points become experimentally distinguishable. This implies that instability reveals some information about the phase space population that was not available at earlier times [14]. The above considerations allow to think chaos as an *information source*. Moreover, the associated rate of generated information can be formulated in a precise way in terms of Kolmogorov-Sinai’s entropy [15], [ 16].

In more recent works, the use of quantifiers based on Information Theory has led to interesting results regarding the characteristics of nonlinear chaotic dynamics, improving the understanding of their associated time series. In particular, the combination of the statistical complexity [17]–[20] and the normalized Shannon entropy, allows for a good distinction between stochastic and chaotic dynamics when incorporating time causal information via the Bandt and Pompe methodology (the permutation probability distribution function (PDF) associated to a time series) [21], [ 22]. This combination generates a graphic tool called the *causality entropy-complexity plane* that was also useful in characterizing dynamical systems from different fields (see [22] and references therein).

The statistical complexity is defined as the product 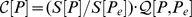 [17] in which, 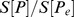 represents the normalized Shannon entropy, 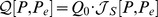 the disequilibrium given in terms of the Jensen-Shannon divergence 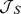 between the PDF associated to the present state of the system (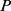) and the uniform PDF (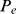), and 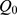 a normalization constant. In the same fashion, Olivares *et al*. [23], [ 24] propose the use of two information quantifiers as measures, namely, the normalized Shannon entropy and the Fisher information combined in the so-called *the causality Shannon-Fisher plane*, finding that stochastic and chaotic dynamics are mapped into different locations.

A close related topic to the Bandt and Pompe permutation PDF is the existence of forbidden patterns. Amigó *et al*. [20], [ 25]–[28] showed that in the case of deterministic chaotic one-dimensional maps not all the possible ordinal patterns can be effectively materialized into orbits, which makes them “forbidden.” In general, one should expect that high-dimensional chaotic dynamical systems (maps) will exhibit forbidden patterns. Indeed, the existence of these *forbidden ordinal patterns* becomes a persistent fact that can be regarded as a “new” dynamical property. Thus, for a fixed pattern-length the number of forbidden patterns of a time series (unobserved patterns) is independent of the series’ length. This independence is not shared by other properties of the series, such as proximity and correlation, which die out with time [26], [ 28].

Stochastic processes could also display forbidden patterns [18], [ 19]. However, in the case of either *uncorrelated* (white noise) or *correlated stochastic processes* (noise with power-law spectrum 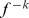 with 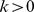, fractional Brownian motion and fractional Gaussian noise) it can be numerically ascertained that *no* forbidden patterns emerge, for a sufficiently time series length. For time series generated by *unconstrained stochastic processes* (uncorrelated processes) every ordinal pattern has the same probability of appearance [25]–[28]. Indeed, if the data set is long enough, all ordinal patterns will eventually appear. In this case, as the number of observations increases, the associated PDF becomes uniform, and the number of observed patterns will depend only on the time series length.

For correlated stochastic processes, the probability of observing a specific individual pattern depends not only on the time series length, but also on the correlation structure [29]. Not observing an ordinal pattern does not qualify it as “*forbidden*”, only as “*missing*”, and this could be due to the time series finite length. A similar observation also holds for the case of real data that always possess a stochastic component due to the omnipresence of dynamical noise [30]–[32]. Thus, “missing ordinal patterns” could be either related to stochastic processes (correlated or uncorrelated) or to deterministic noisy processes (always the case for observational time series).

In particular, Rosso and co-workers recently showed [20] that even when the presence of forbidden patterns is a characteristic of chaotic dynamics, *a minimum pattern-length* is needed to detect their presence. They also showed that the number of forbidden patterns, if they exist, exhibits an exponential behavior with respect to the pattern-length 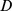, as opposed to the super-exponential behavior described by Amigó and coworkers, valid only for the case 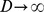 [26], [ 28]. Per contra, in the case of quantifiers evaluated making use of the Bandt and Pompe PDF, a specific behavior emerges in the case of chaotic dynamics that provides a more “robust” distinction between deterministic and stochastic dynamics [17]–[20]. We summarize the learned experience with the use of quantifiers derived from Information Theory, for characterization and distinction between chaotic and stochastic time series, as *the inclusion of the time causality is one of the most important features to consider*.

The use of Visibility Graphs (VG) introduced by Lacasa and co-workers [33], a method that transform a time series into a graph, has also been used with this purpose. Specifically, HVGs, a geometrical simplification of VGs, which is also computationally faster, was applied in the classification and characterization of periodic, chaotic, and onset of chaos dynamics [34], [ 35].

This methodology also incorporates in a natural way the time causality, which is a fundamental component in constructing and assessing Information Theory quantifiers able to distinguish chaos from noise.

Lacasa and Toral [36] studied the discrimination between chaotic, uncorrelated and correlated stochastic time series by using HVG. They conjecture that the node degree distribution of these systems follows an exponential functional of the form 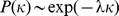, in which 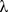 is a positive parameter and 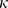 the node degree. They computed analytically the HVG-PDF for the case of uncorrelated noise (white noise) [37], and found the corresponding parameter value 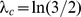. Moreover, they hypothesized that this value corresponds to a central value that separates correlated stochastic (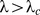) from chaotic dynamics (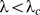).

Even though the methodology works for several chaotic and stochastic systems, we have found several examples for which results diverge from the ones expected.

In this work, we present a methodology able to discriminate between chaotic and stochastic (uncorrelated and correlated) time series by using the HVG methodology together with Information Theory quantifiers. A total of 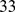 systems are considered; the 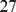 chaotic maps described by Sprott [38], the Schuster map [39], 2 chaotic flows (Lorenz and Rössler chaotic systems) and noises with 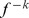, 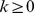 power spectrum (PS) and stochastic time series generated by fractional Brownian motion (fBm) and fractional Gaussian noise (fGn) [17].

Following Olivares *et al*. [23], [ 24] we based our analysis on the so-called Shannon-Fisher information plane (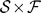) that captures both global and local features of the system’s dynamics. Its horizontal and vertical axis are functionals of the pertinent probability distribution, namely, the normalized Shannon entropy (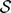) and the normalized Fisher Information measure (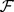). We evaluate these quantifiers for the time series using as PDF the node degree distribution obtained via the horizontal visibility graph. We show that the Shannon-Fisher information plane is able to efficiently represent the different nature of the systems in a planar representation, as well as to distinguish between the different degrees of correlation structures.

As for the organization of this work, the forthcoming Section enumerates and describes the chaotic maps, the chaotic flows, and the stochastic processes considered. Section describes the Horizontal Visibility Graph algorithm and discusses the characterization of the HVG-PDF from a statistical point of view, as well as the methodology implemented in [36] based on the parameter 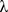. In Section the basis on the Shannon-Fisher plane is detailed, and finally, Section presents our results and discussions. Section 0 concludes the article.

## Materials and Methods

### Chaotic maps, chaotic flows and stochastic processes

#### Chaotic maps

In the present work we consider 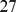 chaotic maps described by Sprott in his book [38] and the Schuster Maps [39] (see Figures S1-S3 in File S1), grouped as follows:

**noninvertible maps:** (1) Logistic map [40]; (2) Sine map [41]; (3) Tent map [42]; (4) Linear congruential generator [43]; (5) Cubic map [44]; (6) Ricker’s population model [45]; (7) Gauss map [46]; (8) Cusp map [47]; (9) Pinchers map [48]; (10) Spence map [49]; (11) Sine-circle map [50].

**dissipative maps:** (12) Hénon map [51]; (13) Lozi map [52]; (14) Delayed logistic map [53]; (15) Tinkerbell map [54]; (16) Burgers’ map [55]; (17) Holmes cubic map [56]; (18) Dissipative standard map [57]; (19) Ikeda map [58]; (20) Sinai map [59]; (21) Discrete predator-prey map [60].

**conservative maps:** (22) Chirikov standard map [61]; (23) Hénon area-preserving quadratic map [62]; (24) Arnold’s cat map [63]; (25) Gingerbreadman map [64]; (26) Chaotic web map [65]; (27) Lorenz three-dimensional chaotic map [66].

We also analyze the Schuster map, a class introduced by Schuster and co-workers [39] that exhibits intermittent signals with chaotic bursts and 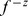 power spectrum (PS). It is defined as:



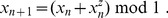
(1)


As the parameter 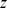 increases, the laminar zone increases in size and the chaotic bursts are less frequent. To generate these maps we use random initial conditions in the interval 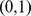 and we consider 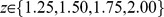.

For noninvertible, dissipative and conservative maps we use the initial conditions and parameter-values detailed by Sprott. The corresponding initial values are given in the basin of attraction for noninvertible maps, near the attractor for dissipative maps, and in the chaotic sea for conservative maps [38]. In the generation process 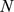 iterations were considered after discarding the first 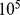. In the case of multi-dimensional maps, we consider all map coordinates. A complete description of these maps can be found in File S1.

#### Chaotic flows

Time series generated by chaotic flows (integration of continuous nonlinear ordinal differential equations) can be also considered. In opposition to the case of chaotic maps, where the sampling period is 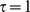, the corresponding value of 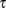 requires careful consideration. *a*) If 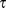 is too small, little information gain accrues between successive sampled values (the values in the corresponding time series will be almost linearly dependent), a *redundancy effect* [67], implying i.e., that the normalized permutation entropy values 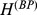 (normalized Shannon entropy evaluated with Bandt-Pompe PDF) “moves” to regions with 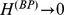. *b*) If 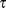 is too large, successive sampled values may became unrelated, an *irrelevance effect* [67]. The normalized permutation entropy values shifts to 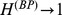 zones. In consequence, time series representative of chaotic flows (chaotic sampled attractors) must be sampled at a specific time 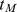, characteristic of the dynamics. Such 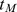 will be the result of an optimal tradeoff between redundancy and irrelevance effects previously mentioned. Two different methodologies based on properties of Information Theory quantifiers evaluated with Bandt-Pompe PDF, have been proposed by Rosso and coworkers. The first one is based on the sample period which maximizes the permutation statistical complexity [68], and the second one, is based on variation of the embedding time used on the attractor’s reconstruction which, in time, gives origin to permutation patterns [69].

An alternative is simplifying the analysis of the corresponding nonlinear differential equations by reducing it to an iterated map of some kind, for instance, a Poincaré map or the time series formed by the minimum values of one variable of the chaotic dynamical system. In the present work, we follow the last option and consider the case of *minimum Lorenz map* [70]. Our results refer to two paradigmatic chaotic systems in a 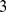-dimensional state space, namely,

#### The Lorenz chaotic attractor [71]



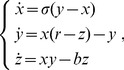
(2)where the pertinent parameters are 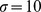, 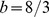, and 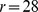, corresponding to a chaotic attractor. The corresponding Lyapunov exponents (base-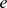) are 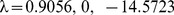 [38].

#### The Rössler chaotic attractor [72]



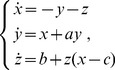
(3)where the parameters used here are 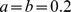 and 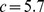, corresponding to a chaotic attractor. The corresponding Lyapunov exponents (base-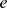) are 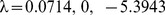 [38].

The corresponding Lorenz minimum map for both chaotic Lorenz and Rössler systems were obtained by integration of the corresponding nonlinear ordinary differential equations (eq. (2) and eq. (3)) using a fourth-order Runge-Kutta method with adaptive stepsize control [73] and integration steps 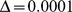 (Lorenz system) and 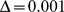 (Rössler system) respectively, with 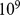 iterations. The minimum values for an orbit with initial value 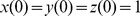 were determined. The first 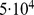 (Lorenz) and 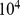 (Rössler) iterations were discharged as transitory. In this way, time series corresponding to the minimum values of 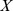-coordinate with at least 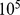 data, were generated.

#### Stochastic processes

The following classical stochastic processes are considered in this work:

**Noises with** 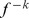 **power spectrum:** These noises are generated as follows [74],

1. By using the Mersenne twister generator [75], the Matlab *RAND* function is used to produce pseudo random numbers in the interval 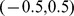 with an almost flat power spectrum (PS), uniform PDF, and zero mean value.
2. The Fast Fourier Transform (FFT) 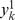 of the time series is obtained and multiplied by 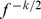 (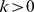), yielding 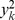;

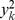
 is symmetrized so as to obtain a real function. The pertinent inverse FFT is obtained after rounding off and truncation. The ensuing time series 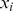 has the desired power-spectrum properties and, by construction, is representative of non-Gaussian noises. In this work we consider noises in the range 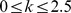, with 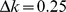.

**Fractional Brownian motion (fBm) and fractional Gaussian noise (fGn):** fBm is the only family of processes which is Gaussian, self-similar, and endowed with stationary increments (see Ref. [76] and references therein). The normalized family of these Gaussian processes, 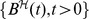, has the following properties: *i*) 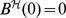 almost surely, *ii*) 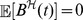 (zero mean), and *iii*) covariance given by



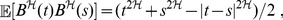
(4)for 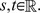 Here 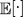 refers to the mean. The power exponent 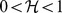 is commonly known as the Hurst parameter or Hurst exponent. These processes exhibit *memory* for any Hurst parameter except for 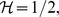 as one realizes from Eq. (4). The 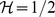 case corresponds to classical Brownian motion and successive motion-increments are as likely to have the same sign as the opposite (there is no correlation among them). Thus, Hurst’s parameter defines two distinct regions in the interval 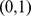. When 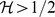, consecutive increments tend to have the same sign so that these processes are *persistent*. On the contrary, for 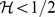, consecutive increments are more likely to have opposite signs *anti-persistent*.

Let us introduce the quantity 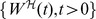 (fBm-“increments”)



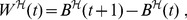
(5)so as to express our Gaussian noise in the fashion



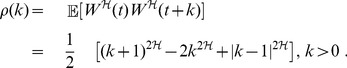
(6)


Note that for 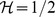 all correlations at nonzero lags vanish and 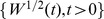 represents *white Gaussian noise*.

The fBm and fGn are continuous but non-differentiable processes (in the classical sense). As non-stationary processes, they do not possess a spectrum defined in the usual sense; however, it is possible to define a *generalized power spectrum* of the form:



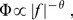
(7)with 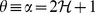, and 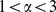 for fBm and; 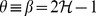, and 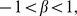 for fGn.

We use the Matlab function “wfbm” that returns a fractional Brownian motion signal with a Hurst parameter 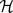 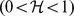 and length 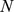 for the generation the fBm and fGn time series. The algorithm was proposed by Abry and Sellan [77], [ 78]. In this work we consider noises in the range 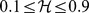.

#### Noise contamination

We attempt to distinguish between stochastic and chaotic dynamics by recourse to an appropriate representation whose starring role is played by quantifiers based in Information Theory combined with Horizontal Visibility Graphs. We deal with well-known models that generate time series according to prespecified rules. This is to be contrasted with the situation posed by real data that always possess a stochastic component due to omnipresent dynamical noise [30]–[32]. Indeed, Wold proved [30] that any (stationary) time series can be decomposed into two different parts. The first (deterministic) part can be exactly described by a linear combination of its own past. The second part is a moving average component of finite order. Hence it may seem superfluous to ask whether a time series generated by natural processes is either deterministic, chaotic, or stochastic. However, having in mind Wold’s theorem [31], [ 32] it makes sense to ask, with respect to the deterministic part (predictable from the past), whether (*i*) it is dominant vis-à-vis the unpredictable stochastic part, or (*ii*) it is of a regular or chaotic nature.

The logistic map constitutes a canonic example, often employed to illustrate new concepts and/or methods for the analysis of dynamical systems. Here we will use the logistic map (full chaotic behavior, 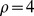) with additive white noise (observational noise) in order to exemplify the behavior of noise contamination over the Information Theory quantifiers evaluated with the PDF-HVG.

The logistic map [38] is a polynomial mapping of degree 2, 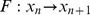, described by the ecologically motivated dissipative system represented by the first-order difference equation



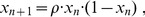
(8)with 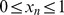 and 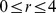.

Let 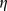 be the observational white noise. We generated it by using the Mersenne twister generator [75] through the Matlab *RAND* function, which produces pseudo random numbers in the interval 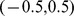 with an almost flat power spectrum (PS), uniform PDF, and zero mean value. We consider time series of the form 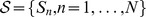 generated by the discrete system:



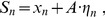
(9)in which 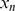 is given by the full chaotic logistic map, and 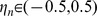 is the additive noise with amplitude 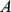. We consider time series with 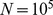 data and noise amplitudes in the range 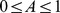 with 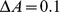.

### Horizontal Visibility Graph

The horizontal visibility graph (HVG) [37] is a geometrical simplification of the visibility graph (VG) [33] that maintains the inherent characteristics of the transformed time series and incorporates in a natural way its time causality.

By construction, the HVG transforms a time series into a graph, in which each node corresponds to a point in the time series, will be connected considering the following criterion:

Let 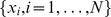, be a time series of 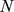 data. Two nodes 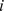 and 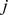 in the graph are connected if it is possible to trace a horizontal line in the time series linking 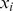 and 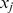 not intersecting intermediate data height, fulfilling: 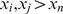 for all 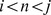.

**Figure 1:**
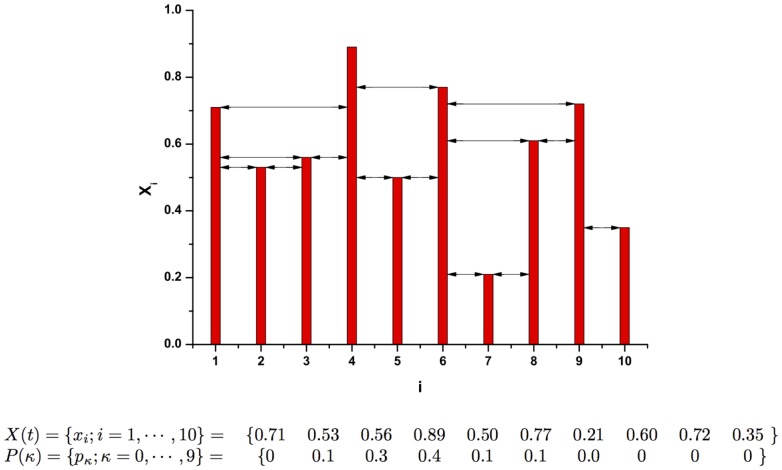
Horizontal Visibility Graph method applied to the time series 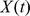. 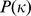
 denotes the node degree distribution of the obtained graph (HVG-PDF).

Note that the HVG preserves the time causality of the original series where each node sees at least its nearest neighbors. Another important feature of the HVG is the invariance under affine transformations, as its visibility is not modified under rescaling of horizontal and vertical axes, as well as under horizontal and vertical translations. Some other interesting properties are discussed in [37], [ 79]. The last work focus on how some topological properties of the HVG transformed from Fractional Brownian motion change depending on the different values of the Hurst exponent. An example of a time series and its associated node degree distribution based on HVG is given in Figure 1.

#### The 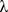 rule

Lacassa and Toral [36] propose that chaotic and stochastic time series map into a graph with an exponential node degree distribution 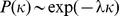. The 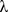 parameter is computed by adjusting, using the least square method, a straight line being 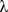 its slope. The linear scaling region considered by Lacassa and Toral is 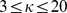 or 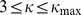 (if 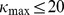) for stochastic processes; and 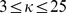 or 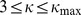 (if 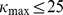) for chaotic ones. The parameter 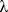 characterizes chaotic processes when 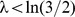, uncorrelated noises for 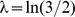 and correlated noises when 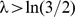.

**Figure 2:**
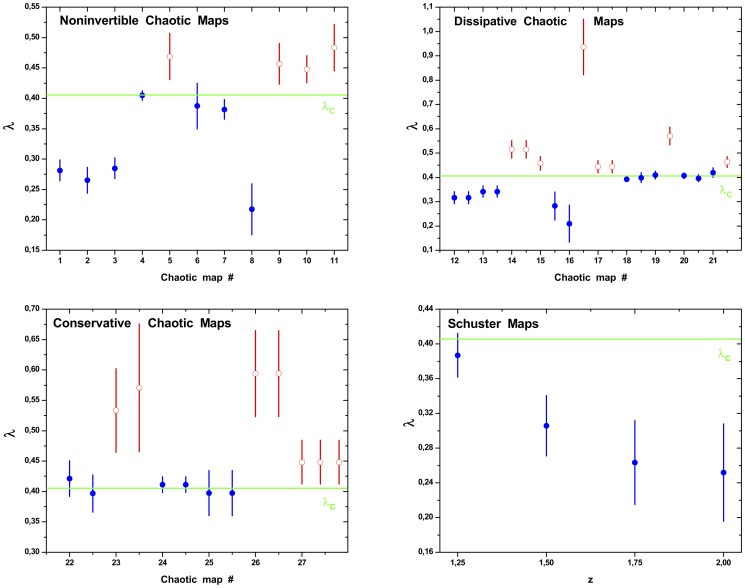
Confidence intervals for 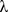 values for Noninvertible, Dissipative, Conservative and Schuster Chaotic Maps. The values were obtained following the methodology proposed by Lacasa *et al.*. Symmetric confidence intervals at the 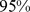 confidence level were obtained for the 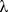 parameter assuming the Gaussian model, a linear structure for the regression and independent zero-mean errors. The horizontal line represents the value of 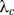 corresponding to white noise (uncorrelated stochastic dynamics). The list of names for each map is the same given in Sec.. Full circles (blue) are in agreement with Lacassa and Toral [36] proposal rule. Empty circles (red) not.

**Figure 3:**
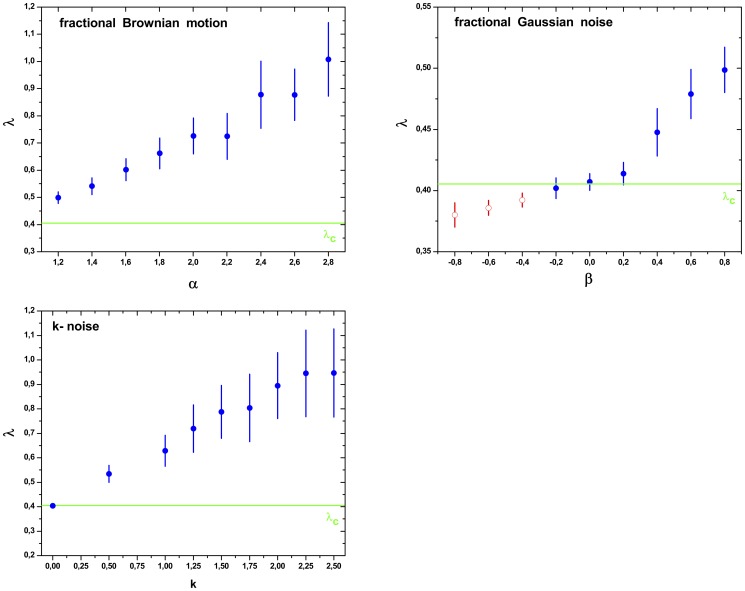
Parameter 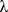 values of HVG-PDF 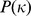 for fBm, fGn and noise with 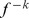 power spectrum time series with total length of 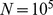 data. The 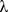 values were obtained following the methodology proposed by Lacasa *et al.*: from the graph 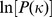 versus 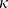, the 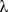 parameter was computed by adjusting using the least square method, a straight line being 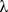 its slope. The linear scaling region considered in all cases is 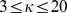, or 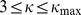 (if 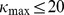). Symmetric confidence intervals at the 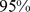 confidence level were obtained for the 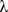 parameter assuming the Gaussian model, a linear structure for the regression and independent zero-mean errors. The horizontal line represents the value of 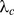 corresponding to white noise (uncorrelated stochastic dynamics) Full circles (blue) are in agreement with Lacassa and Toral [36] proposal rule. Empty circles (red) not.

In the same fashion, we have computed the node degree distribution 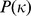 of the HVG for all the systems described in Section for series of 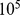 data length. Symmetric confidence intervals at the 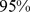 confidence level were obtained assuming the Gaussian model, a linear structure for the regression and independent zero-mean errors. They will be denoted between brackets after the point estimation of 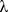. The results are shown in Figures 2 and 3 for all studied chaotic maps and stochastic dynamics, respectively. For the chaotic flows we obtain 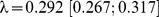 and 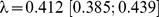 for the Lorenz system (coordinate X) and Rössler system (coordinate X), respectively. It is possible to see from these figures that several chaotic and stochastic systems follow the above mentioned rule; however, we have found others that do not, like the Rössler chaotic system (coordinate X). Examples of chaotic maps for which 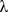 is larger than 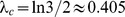 (see Fig. 2 open circles) correspond to: (5) cubic map, (9) Pinchers map, (10) Spence map, (11) sine-circle map, (14) delay logistic map, (15) Tinkerbell map (X), (16) Burger’s map (Y), (17) Holmes cubic map, (19) Ikeda map (Y) (21) discrete predator-prey map (Y), (23) Hénon area-preserving quadratic map, (26) chaotic web map, (27) Lorenz three-dimensional chaotic map, (see also Table S1 in File S1). Stochastic processes for which 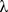 is smaller than 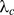 (see Fig. 3 open circles) correspond to fGn with 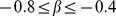.

Considering the case of the logistic map with 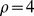 (fully developed chaotic dynamics) contaminated with additive noise (noises with uniform PDF and different amplitudes, 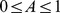)– see Section, the parameter 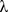 increases between 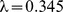 for 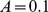 to 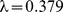 for 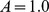, limited for the values 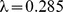 for 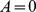 (logistic map) and 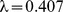 for pure noise. One is able to differentiate between chaotic dynamics contaminated with noise, and pure stochastic dynamics.

**Figure 4:**
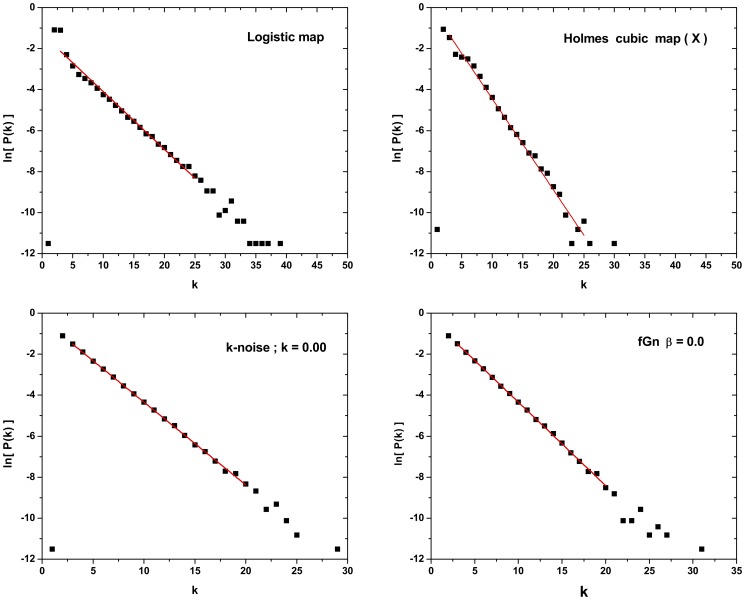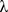-value determination: examples of analyzed dynamical systems where a good linear scaling region was found. For the Holmes cubic map (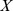), however, even having a good fitting, the 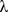-value obtained is greater than 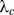 which not satisfied the chaotic distinction suggested by Lacasa and Toral [36]. In all cases, time series with 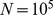 are considered, and linear scaling regions are defined by 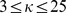 for chaotic and 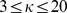 for stochastic time series.

Some important issues to be discussed are:

**Scaling zone:** Several systems present a well defined linear scaling region allowing a good linear fitting to obtain 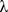. Examples are the Logistic map, Holmes cubic map (X), a 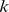-noise with 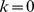 and a fBm with 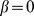 presented in Figure 4. However, we must point out that the fact of having a well scaling region does not guarantee the satisfaction of the 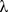 rule. See for instance the Holmes cubic map (X) that present a clear linear scaling region, however 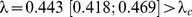, contradicting the hypothesis.

**Figure 5:**
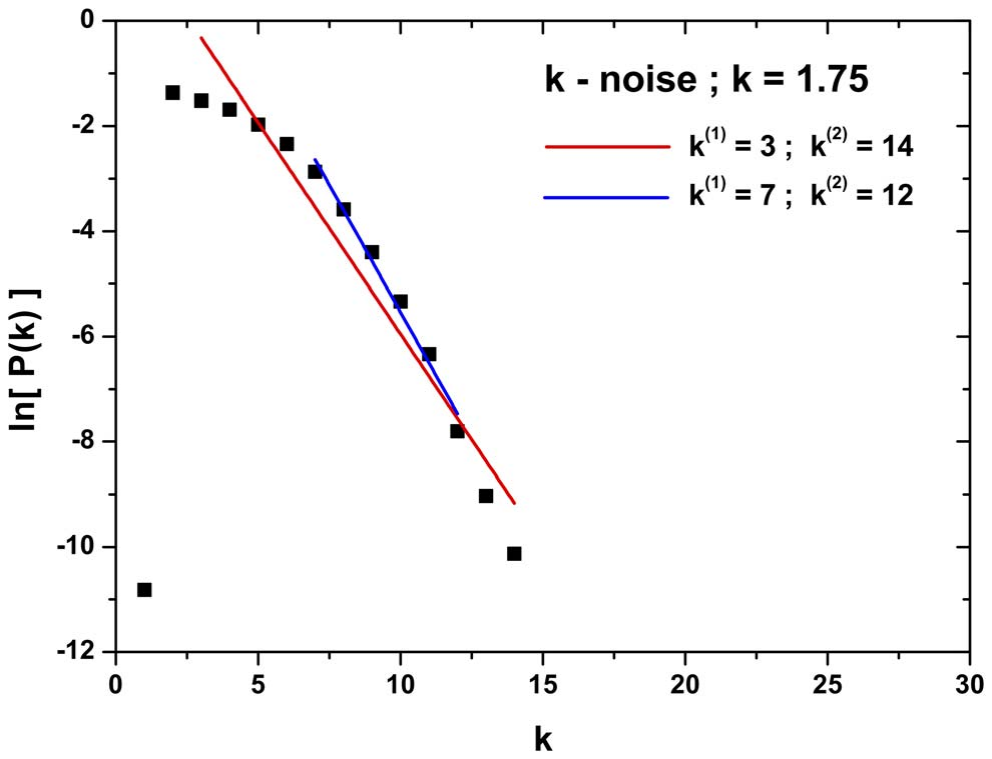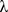-value determination in the case of time series generated by stochastic dynamics with 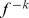 power spectrum with 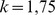. Time series with 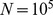 data. Two different linear scaling zones: *a*) 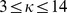 given 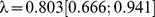; and *b*) 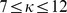 given 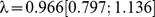. Note that the slope of the straight line change significantly.

Another important point is the selection of the scaling zone, as the inclusion or exclusion of a few points in the extremes of the PDF may drastically change the 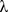 value. Figure 5 shows the effect of selecting different scaling zones for a stochastic process with 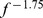 PS . If the scaling zone is defined in the node degree interval 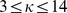, 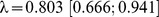, however, if the scaling zone is redefined for the interval 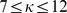, 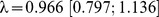, which represent a variation of 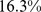.

**Figure 6:**
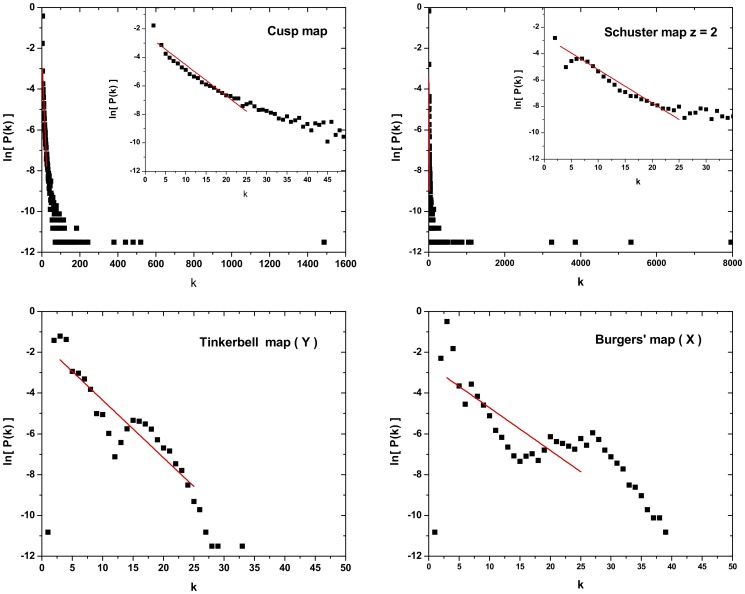
Cases with bad 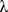-value determination: *a*) Cusp map and Schuster map with 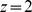, the associated HVG-PDF present heavy tail making difficult to define an unique linear scaling zone representative of all the data. *b*) Tinkerbell map (Y) and the Burger’s map (X) for which it is impossible to define an unique linear scaling zone, and in consequence the hypothesis of an exponential behavior cannot be confirmed. Time series with 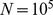 data are considered.

**Heavytailedness:** The definition of an unique linear scaling zone is a difficult task for systems with a heavy tailed PDF. Note that, when defining a scaling zone, important information contained in the tails may be lost. Examples of systems with heavy tailed PDFs are the Cusp and the Schuster maps (see Fig. 6).

**Nonexponential behavior:** Some systems present PDFs with no linear scaling zone, in consequence, the hypothesis of an exponential behavior cannot be confirmed. See for instance, the Tinkerbell map (Y) and the Burger’s map (X) in Fig. 6.

In Table S1 of File S1 readers can find the 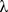 values with the corresponding confidence intervals, the coefficient of determination 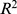, as well all the corresponding plots for all the dynamical systems analyzed in this work (Figures S4-S12).

#### Skewness and kurtosis

Given a one-dimensional probability distribution 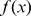 with 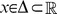, the usual spread measure is the variance 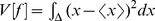. The variance measures the (quadratic) variability around the mean. This property makes the variance (or its square root, the standard deviation) particularly useful for smooth unimodal distributions. Other interesting quantifiers based on higher moments order are the skewness (a third order moment measure) and the kurtosis (which depends on the fourth order moment). The skewness measures the asymmetry, while the kurtosis describes the relative “peakedness” of the density with respect to the Gaussian law. Kurtosis is a sign of “flattening” or “peakedness” of a distribution.

The usual skewness and kurtosis are of limited use and interpretability when dealing with asymmetric distributions, as is the case of the node degree distribution HVG-PDF 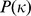, which is always non-negative.

Among the many alternatives available in the literature, for skewness and kurtosis evaluation, Brys *et al.* [80] employ with success the information provided by the quantiles. In particular, we will see that an alternative measure of kurtosis is able to describe the different heavytailedness of the observed node degree distribution HVG-PDF.

Consider 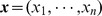 a sample of 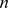 real values. The sample quantile of order 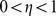 is 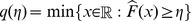 and 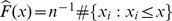, where 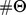 denotes the cardinality of the set 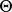, is the sample cumulative distribution function also known as empirical function. Quantile-based measures of skewness and kurtosis can be defined as



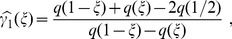
(10)


and



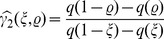
(11)respectively, where 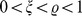 are arbitrary quantiles.

The values for two reference distributions were computed analytically with 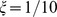 and 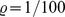. For the standard exponential distribution with probability density function 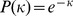, 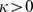 they are:



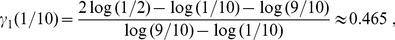
(12)


and



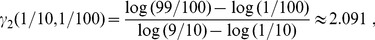
(13)and for the node degree distribution under white noise, whose probability function is 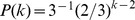, 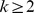 [33], [ 36], they are 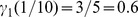 and 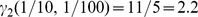.

Table 1 shows the values of lambda (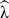), skewness (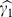) and kurtosis (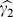) for several noises and chaotic maps.

**Table 1:** Dynamical systems and their statistical quantifiers skewness (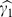), kurtosis (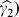 evaluated for 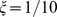 and 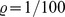.

System	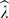	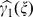	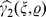
Exponential	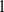	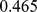	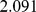
White Noise	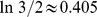	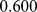	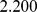
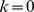 Noise	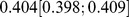	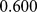	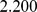
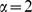 fBm	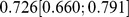	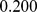	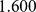
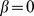 fGn	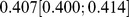	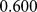	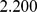
Logistic map	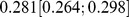	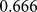	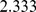
Cusp map	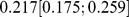	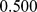	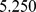
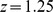 Schuster	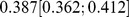	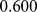	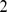
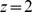 Schuster	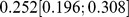	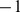	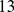

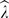
 is the obtained parameter value (with confidence value interval) of exponential functional form proposed by Lacasa and Toral for the HVG-PDF [36]). Time series with 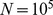 data are considered.

**Figure 7:**
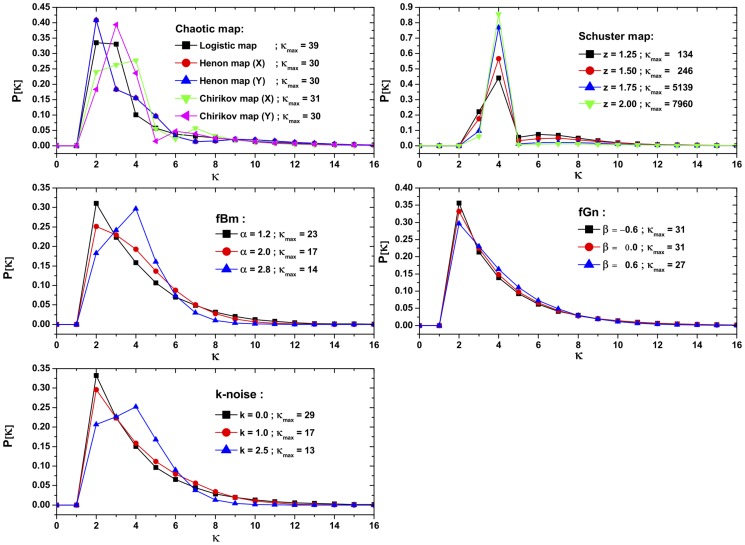
Examples of HVG-PDF for some chaotic and stochastic systems. Only 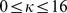 are displayed. Note that the corresponding cut-offs (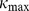) are also shown. The length of the time series is 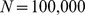.

It is worth noticing that several chaotic maps present high kurtosis values indicating a heavy tailed PDF, showing the importance of using a quantifier that considers the entire available data. Fig. 7 displays examples of HVG-PDF of several chaotic and stochastic systems. Note that, for some systems, the HVG-PDFs do not present an exponential behavior. Readers can find the results for all the systems considered in Table S1 of File S1.

### The Shannon-Fisher information plane

To avoid the subjectivity of choosing the scaling zone in which the parameter 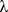 is computed and, consequently, the sensitivity of this methodology, we propose a tool in which no information is lost, as the entire PDF is used and the relation between global and local features of the systems is captured. The Shannon-Fisher information plane (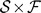) firstly introduced by Vignat and Bercher [81] is a planar representation in which the horizontal and vertical axes are functionals of the pertinent probability distribution, namely, the Shannon Entropy 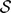 and the Fisher Information measure 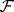, respectively. This tool is a convenient way to represent in the same information plane global and local aspects of the PDFs associated to the studied system. In this work the PDFs are obtained through the horizontal visibility graph methodology [33].

Given a continuous probability distribution function (PDF) 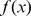 with 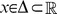 and 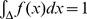, its *Shannon Entropy* [82] is



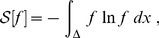
(14)a measure of “*global*” character that it is not too sensitive to strong changes in the distribution taking place on small regions of the support 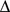.

Such is not the case with *Fisher’s Information Measure* (FIM) 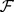 [83]–[85], which constitutes a measure of the gradient content of the distribution 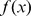, thus being quite sensitive even to tiny localized perturbations. It reads



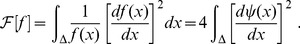
(15)


FIM can be variously interpreted as a measure of the ability to estimate a parameter, as the amount of information that can be extracted from a set of measurements, and also as a measure of the state of disorder of a system or phenomenon [85]. In the previous definition of FIM (Eq. (15)) the division by 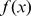 is not convenient if 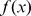 becomes too small to be adequately computed. Such issue is avoided using probability amplitudes 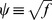 [84], [ 85]. The gradient operator significantly influences the contribution of minute local 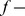
variations to FIM’s value. Accordingly, this quantifier is called a “*local*” one [85].

Let now 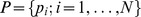 be a discrete probability distribution, with 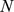 the number of possible states of the system under study. The concomitant problem of information-loss due to discretization has been thoroughly studied (see, for instance, [86]–[88], and references therein) and, in particular, it entails the loss of FIM’s shift-invariance, which is of no importance for our present purposes [23], [ 24]. In the discrete case, we define a “normalized” Shannon entropy as



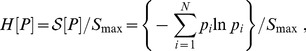
(16)where the denominator 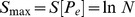 is the Shannon entropy attained by a uniform probability distribution 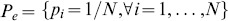. For the FIM we take the expression in terms of real probability amplitudes as starting point, then a discrete normalized FIM convenient for our present purposes, is given by



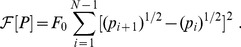
(17)


It has been extensively discussed that this discretization is the best behaved in a discrete environment [89]. Here the normalization constant 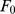 Reads



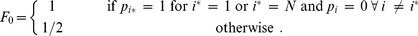
(18)


## Results and Discussion

**Figure 8:**
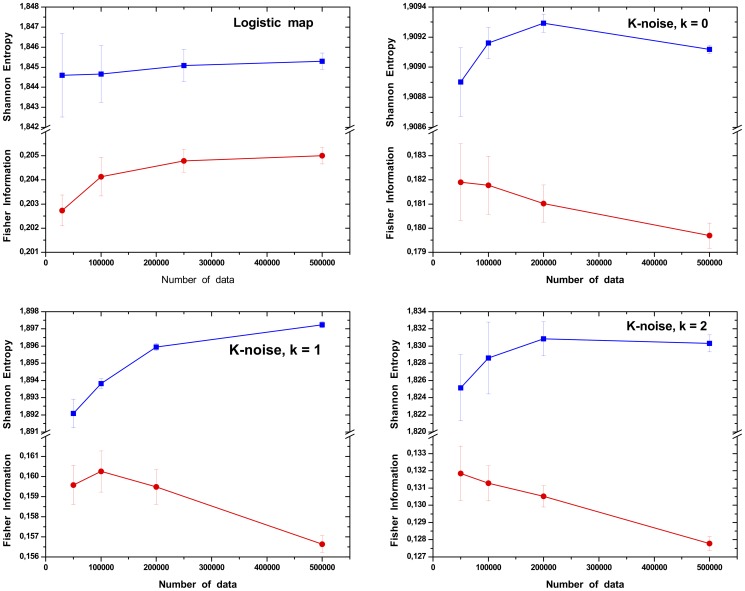
Study of the effect of the series length on the Information Theory quantifiers. The dynamical systems here considered are the Logistic map and noises with 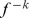 power spectrum, for 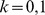 and 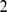.

In order to study the stability of the forthcoming results, we first analyze the dependency of the Information Theory quantifiers with the size of the time series. For this experiment we consider time series with different length sizes, varying from 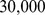 to 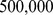 values. As it can be seen in Figure 8, the Fisher Information and the Shannon entropy rapidly converge to stable values. For example, for the cases depicted in Figure 8, the order of magnitude of the percentage variations of the mean value for times series with 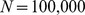 and 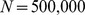 are between 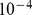 and 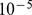. For that reason all experiments consider time series with 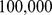 values. The Fisher Information and the Shannon Entropy are computed for all systems presented in Section. Note that, as expected, bi-dimensional maps presenting one delayed coordinate (i.e.: delay logistic map), have identical quantifier values for both time series coordinates.

**Figure 9:**
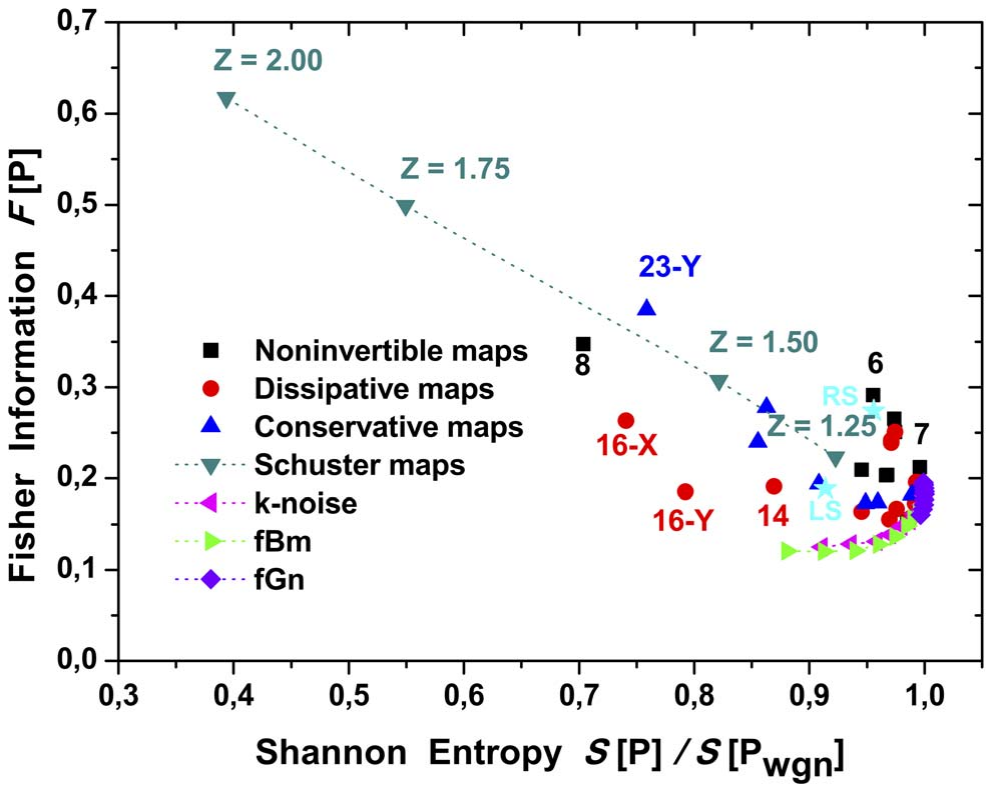
Representation on the Shannon-Fisher plane, 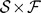, for all dynamical systems. The quantifiers were evaluated with the HVG-PDF from time series length 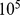. The stars (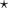) represent the obtained values for chaotic flows (RS: Rössler system (X-coordinate), and LS: Lorenz system (X-coordinate)).

**Figure 10:**
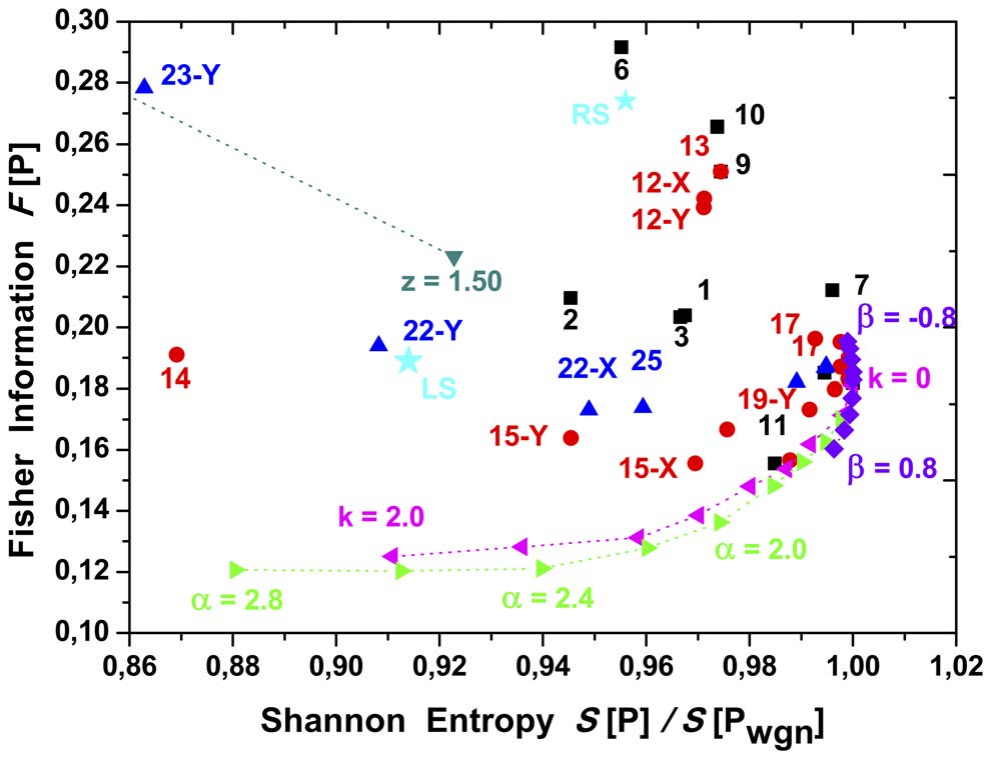
Shannon-Fisher plane, 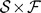 zoom, see Fig. 9.

**Figure 11:**
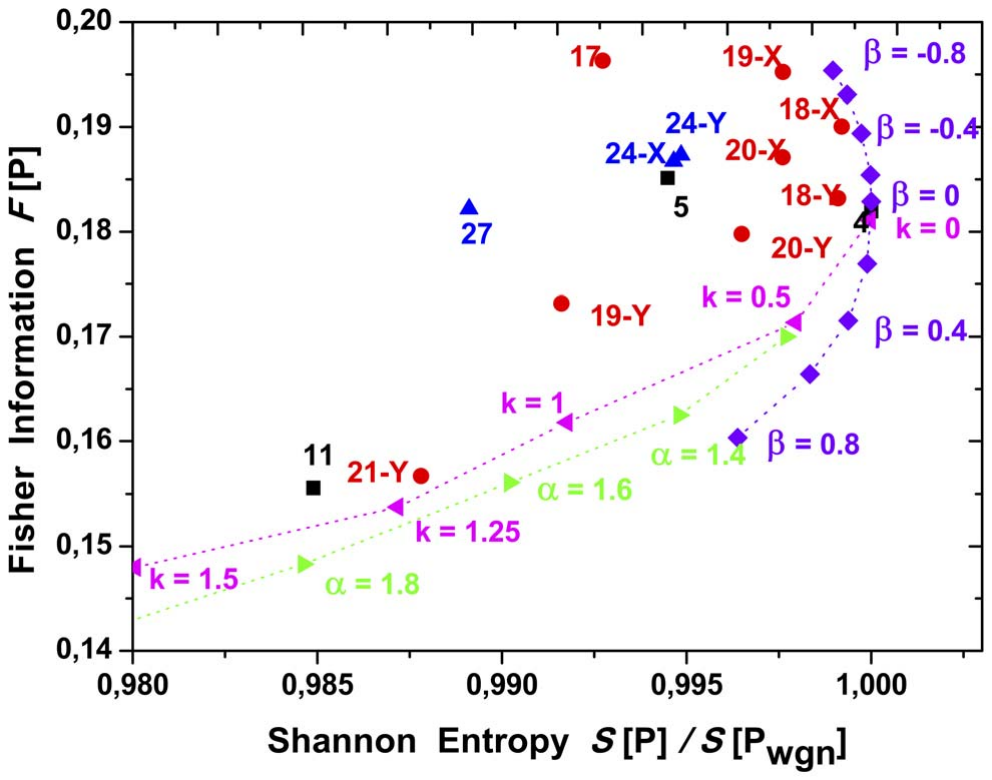
Shannon-Fisher plane, 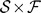 zoom, see Fig. 9.

Results are depicted in Figures 9, 10 and 11. The Shannon entropy values are normalized with its maximum value for 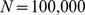, that corresponds to the entropy of the gaussian white noise (fGn for 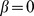, 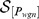). In this work we change the classical 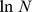 normalization of the Shannon entropy to facilitate the comparison of results when using different time-series lengths. The normalization through 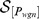 reaches stable values while the normalization through 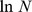 results in decreasing values as the time-series length increases.

One interesting observation is the fact that the Fisher Information (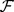) decreases with the strength of correlation in noises. The degree distribution corresponding to noises with lower correlation presents high peaks as well as long tails, almost flat for the white noise. As correlations get stronger, the peaks decrease and tails get shorter. For the uncorrelated situation (white noise), the strong contribution of the long and flat tail, even having the highest peak, makes the shape of the distribution more uniform. This effect can be seen in Figure 7 as well as in Table S1 for noises with 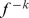 power spectrum.

The statistical complexity, an Information theory quantifier based on the relation between the normalized Shannon entropy and the Jensen-Shannon divergence, was previously used to successfully distinguish stochastic from chaotic dynamics [17], [ 22]. However, when extracting the PDF of the system through HVG, this quantifier presents poor results.

Graphs obtained by applying HVG present very short tail distributions. Thus, when considering time series long enough to capture the dynamics of the systems, the Jensen Shannon divergence cannot clearly discriminate between different systems degree distributions due to the high number of components with 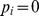. As a direct consequence, the statistical complexity will convey limited new information. The use of the Fisher information measure greatly complements the Shannon entropy as it brings local insights of the degree distribution. Therefore, the 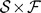 plane allows us to map global and local information describing the nature and similarities of the systems.

The Fisher Information is sensitive to small fluctuations. From Eq. (17), it is possible to see that bigger differences in consecutive 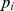 values of the distribution 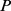, result in higher values of 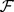. In this case, the higher peaks in the degree distributions, that correspond to lower correlation values, represent the main contribution to 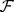. The extra terms present in the long tail, even contributing with small values, still increase the value of 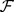. For that reason, the lowest value of 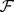 corresponds to the noise with strongest correlation structure (fBm with 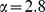), see Figure 11. That is not the case of the Shannon entropy 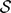, which is not sensitive to small fluctuations. The Shannon Entropy presents its highest value for noises with the smallest correlation (white noises, gaussian and non-gaussian) and, as correlation structures get stronger, 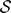 decreases.

The planar localization in the Shannon-Fisher information plane 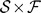, gives interesting information about the relation between the systems. Noises appear to be organized as a frontier, from which all chaotic maps concentrate. As it was previously shown, the frontier is stable regarding the size of the times series length.

Note that some chaotic maps are located nearby the noise “frontier” in the 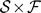 plane (see Figure 11). These maps are: the linear congruential generator (4), the dissipative standard map (18), and the Sinai map (20). They present high 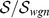 values, 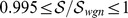 and low 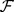 values, 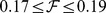. This planar localization can be understood, as these maps present a stochastic like dynamical behavior when represented in a two dimensional plane. However, when represented in higher dimensional planes, planar structures appear denoting their chaotic behavior.

The use of the 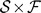 plane can shed light on the underlying system’s structure. For example, the Schuster maps display a linear behavior in the 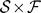 plane when varying the 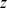 parameter. Wider laminar regions (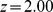) generate a greater number of nodes with lower degree values. At the same time nodes located in the extremes of a laminar region posses higher degree. As the parameter 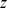 decreases, the laminar structures get thinner, reducing the number of nodes with higher degree value. This fact positioned the Schuster systems far from the frontier as 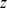 increases as can be seen in Figure 9.

**Figure 12:**
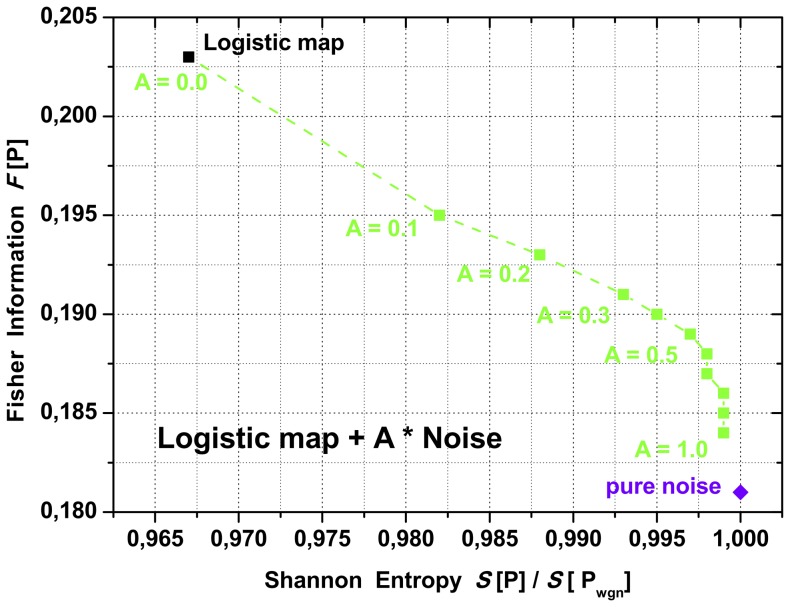
Shannon-Fisher plane, 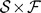, for the logistic map (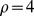) contaminated with additive noise with uniform PDF and amplitude 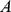. Time series with 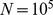 data are considered.

Figure 12 portrays the results obtained after an additive noise contamination (noise with uniform PDF and with different amplitudes, 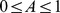) to the logistic map with 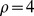 (fully developed chaotic dynamics) – see Section – in the plane 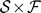. It is easy to see how both quantifiers are able to capture the increase of the noise amplitude, mapping the systems from the original logistic map localization (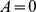) towards the region of pure noise when the noise amplitude increases without overlapping the pure noise planar localization. In Table S1 in File S1 can be found a detailed description of the results.

## Conclusions

This work is divided in two parts; the former includes a thorough numerical analysis to test Lacasa and Toral methodology [36], for several chaotic, stochastic and noise-contaminated systems. The latter presents a methodology based on the HVG combined with Information Theory quantifiers.

The first analysis revealed that the use of the slope of the logarithm of the degree distribution of the HVG obtained from the systems time series, fails in properly divide their nature. The method is highly sensitive to the selection of the scaling zone to compute 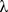, which is in some cases a non-trivial task. The non-exponential behavior and the heavytailedness of the degree distribution make the method dependent to external adjustments. Nevertheless, the HVG itself shows the ability to capture and maintain the intrinsic features of the systems.

In the second part of this manuscript, we propose the use of the Horizontal Visibility Graph in combination with the Shannon entropy and the Fisher information measure as a methodology to study dynamical systems. Several chaotic (maps and flows) and correlated noises were considered for an exhaustive analysis. The arrangement of the results in the 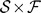 plane shows that this novel tool is able to capture features that reveal the nature governing the system. The 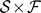 plane exposes the intrinsic features of a system by positioning it in a planar representation, conveniently combining global and local aspects of the PDF under study.

We have presented extensive numerical evidence and have contrasted the characterization of deterministic chaotic, noisy-stochastic dynamics, and chaotic systems contaminated with additive noise of different amplitudes, as represented by time series of finite length. Surprisingly enough, one just has to look at the different planar locations of the two dynamical regimes. The planar location is able to tell us whether we deal with chaotic or stochastic time series.

We claim that the presented methodology can be applied to systems of any dimension. However, the sampling time which capture the correct chaotic dynamics and length of their representative time-series merit a specific analysis for high dimensional systems. Also, the analysis of noise contamination requires a deep and thorough exploration including different types of noises. Works in this direction are in progress.

## Supporting Information

File S1 **Supplemenatry material.**
(PDF)
