# Supplementary material for: Distinguishing Noise from Chaos: Objective versus Subjective Criteria Using Horizontal Visibility Graph
Source: PLoS One. 2014 Sep 23;9(9):e108004. doi: 10.1371/journal.pone.0108004 (PMC4172653; doi:10.1371/journal.pone.0108004)
Supplement: File S1 — Supplemenatry material. (PDF) [file pone.0108004.s001.pdf]

Supplementary Material: Distinguishing noise from chaos: objective versus subjective criteria using visibly graph.

Martín Gómez Ravetti [1,5], Laura C. Carpi [2], Bruna Amin Gonçalves[1,6], Alejandro C. Frery [2], Osvaldo A. Rosso [3,4]

1 Departamento de Engenharia de Produção, Universidade Federal de Minas Gerais.
Av. Antônio Carlos, 6627, 31270-901 Belo Horizonte, Minas Gerais, Brazil.
2 Laboratório de Computação Científica e Análise Numérica (LaCCAN),
Universidade Federal de Alagoas.
BR 104 Norte km 97, 57072-970 Maceió, Alagoas, Brazil.
3 Instituto de Física, Universidade Federal de Alagoas.
BR 104 Norte km 97, 57072-970 Maceió, Alagoas - Brazil.
4 Instituto Tecnológico de Buenos Aires (ITBA),
Av. Eduardo Madero 399 (C1106ACD), Ciudad Autónoma de Buenos Aires, Argentina.
5 Departament de Física Fonamental, Universitat de Barcelona.
08028 Barcelona, Spain.
6 Instituto Politécnico. Centro Universitário UNA.
Av. Raja Gabaglia 3950, predio 1, 30350-540, Belo Horizonte, Minas Gerais, Brazil.
$*$ E-mail: martin.ravetti@dep.ufmg.br

1 Considered chaotic systems and their parameters

1.1 Noninvertible maps

The nonivertible maps considered, their parameters, initial condition and the corresponding Lyapunov exponent, are listed below (see Ref. Sprott (2003)). The Fig. 1 show X_{n+1} versus X_n for these one-dimensional maps.

- *Logistic map :*

$$X_{n+1} \ = \ \rho \ X_n \ (\ 1 - X_n \ ) \ . \tag{1}$$

  Parameter value: $\rho = 4$; initial condition: $X_0 = 0.1$; Lyapunov exponent: $\lambda = \ln 2 = 0.693147181\ldots$
  Ref: May (1976).

- *Sine map :*

$$X_{n+1} \ = \ A \ \sin \pi X_n \ . \tag{2}$$

  Parameter value: $A = 1$; initial condition: $X_0 = 0.1$; Lyapunov exponent: $\lambda \simeq 0.689067$.
  Ref: Strogatz (1994).

- *Tent map :*

$$X_{n+1} \ = \ A \ \mathrm{Min}\{ \ X_n, 1 - X_n \ \} \ . \tag{3}$$

  Parameter value: $A = 2$; initial condition: $X_0 = 1/\sqrt{2}$; Lyapunov exponent: $\lambda = \ln|A| = 0.693147181\ldots$
  Ref: Devaney (1989).

- *Linear congruential generator :*

$$X_{n+1} \;=\; A\;X_n + B\;X_n \qquad (\;\mathrm{Mod}\;C\;)\;. \tag{4}$$

Parameter values: $A = 7141$, $B = 54773$, $C = 259200$; initial condition: $X_0 = 0$; Lyapunov exponent: $\lambda = \ln|A| = 8.873608101\ldots$
Ref: Knuth (1997)

- *Cubic map :*

$$X_{n+1} \;=\; A\;X_n\;(\;1 - X_n^2\;)\;. \tag{5}$$

Parameter value: $A = 3$; initial condition: $X_0 = 0.1$; Lyapunov exponent: $\lambda \simeq 1.0986122883$.
Ref: Zeng *et al.* (1985).

- *Ricker's population model :*

$$X_{n+1} \;=\; A\;X_n\;e^{-X_n}\;. \tag{6}$$

Parameter value: $A = 20$; initial condition: $X_0 = 0.1$; Lyapunov exponent: $\lambda \simeq 0.384846$.
Ref: Ricker (1954).

- *Gauss map :*

$$X_{n+1} \;=\; 1/X_n \qquad (\;\mathrm{Mod}\;1\;)\;. \tag{7}$$

Initial condition: $X_0 = 1$; Lyapunov exponent: $\lambda \simeq 2.373445$.
Ref: van Wyk and Steeb (1997).

- *Cusp map :*

$$X_{n+1} \;=\; 1 - A\;\sqrt{|\;X_n\;|}\;. \tag{8}$$

Parameter value: $A = 2$; initial condition: $X_0 = 0.5$; Lyapunov exponent: $\lambda = 0.5$.
Ref: Beck and Schlögl (1995).

- *Pinchers map :*

$$X_{n+1} \;=\; |\;\tanh S\;(\;X_n - C\;)\;|\;. \tag{9}$$

Parameter values: $S = 2$, $C = 0.5$; initial condition: $X_0 = 0$; Lyapunov exponent: $\lambda \simeq 0.467944$.
Ref: Potapov and Ali (2000).

- *Spence map :*

$$X_{n+1} \;=\; |\;\ln X_n\;|\;. \tag{10}$$

Initial condition: $X_0 = 0.5$; Lyapunov exponent: $\lambda \to \infty$.
Ref: Shaw (1981).

- *Sine–circle map :*

$$X_{n+1} \;=\; X_n + \Omega - \frac{K}{2\pi}\;\sin 2\pi X_n \qquad (\;\mathrm{Mod}\;1\;)\;. \tag{11}$$

Parameter values: $\Omega = 0.5$, $K = 2$; initial condition: $X_0 = 0.1$; Lyapunov exponent: $\lambda \simeq 0.353863$.
Ref: Arnold (1965).

1.2 Dissipative maps

The dissipative maps considered, their parameters, initial condition and the corresponding Lyapunov exponents, are listed below (see Ref. Sprott (2003)). The Fig. 2 show X_n versus Y_n for these two-dimensional maps.

- *Hénon map :*

$$\left\{ \begin{array}{lcl} X_{n+1} & = & 1 - a\ X_n^2 + b\ Y_n \\ Y_{n+1} & = & X_n \end{array} \right. . \tag{12}$$

Parameter values: $a = 1.4$, $b = 0.3$; initial conditions: $X_0 = 0$, $Y_0 = 0.9$; Lyapunov exponents: $\lambda_1 \simeq 0.41922$, $\lambda_2 \simeq -1.62319$.
Ref: Hénon (1976).

- *Lozi map :*

$$\left\{ \begin{array}{lcl} X_{n+1} & = & 1 - a \mid X_n \mid + b\ Y_n \\ Y_{n+1} & = & X_n \end{array} \right. . \tag{13}$$

Parameter values: $a = 1.7$, $b = 0.5$; initial conditions: $X_0 = -0.1$, $Y_0 = 0.1$; Lyapunov exponents: $\lambda_1 \simeq 0.47023$, $\lambda_2 \simeq -1.16338$.
Ref: Lozi (1978).

- *Delayed logistic map :*

$$\left\{ \begin{array}{lcl} X_{n+1} & = & A\ X_n\ (\ 1 - Y_n\ ) \\ Y_{n+1} & = & X_n \end{array} \right. . \tag{14}$$

Parameter values: $A = 2.27$; initial conditions: $X_0 = 0.001$, $Y_0 = 0.001$; Lyapunov exponents: $\lambda_1 \simeq 0.18312$, $\lambda_2 \simeq -1.24199$.
Ref: Aronson *et al.* (1982).

- *Tinkerbell map :*

$$\left\{ \begin{array}{lcl} X_{n+1} & = & X_n^2 - Y_n^2 + a\ X_n + b\ Y_n \\ Y_{n+1} & = & 2\ X_n\ Y_n + c\ X_n + d\ Y_n \end{array} \right. . \tag{15}$$

Parameter values: $a = 0.9$, $b = -0.6$, $c = 2$, $d = 0.5$; initial conditions: $X_0 = 0$, $Y_0 = 0.5$; Lyapunov exponents: $\lambda_1 \simeq 0.18997$, $\lambda_2 \simeq -0.52091$.
Ref: Nusse and Yorke (1994).

- *Burgers' map :*

$$\left\{ \begin{array}{lcl} X_{n+1} & = & a\ X_n^2 - Y_n^2 \\ Y_{n+1} & = & b\ Y_n + X_n\ Y_n \end{array} \right. . \tag{16}$$

Parameter values: $a = 0.75$, $b = 1.75$; initial conditions: $X_0 = -0.1$, $Y_0 = 0.1$; Lyapunov exponents: $\lambda_1 \simeq 0.12076$, $\lambda_2 \simeq -0.22136$.
Ref: Whitehead and Macdonald (1984).

- *Holmes cubic map :*

$$\left\{ \begin{array}{lcl} X_{n+1} & = & Y_n \\ Y_{n+1} & = & -b\ X_n + d\ Y_n - Y_n^3 \end{array} \right. . \tag{17}$$

Parameter values: $b = 0.2$, $d = 2.77$; initial conditions: $X_0 = 1.6$, $Y_0 = 0$; Lyapunov exponents: $\lambda_1 \simeq 0.59458$, $\lambda_2 \simeq -2.20402$.
Ref: Holmes (1979).

- *Dissipative standard map :*

$$\begin{cases} X_{n+1} = X_n + Y_{n+1} & (\text{ Mod } 2\pi \text{ }) \\ Y_{n+1} = b\, Y_n + k\ \sin(X_n) & (\text{ Mod } 2\pi \text{ }) \end{cases} . \tag{18}$$

Parameter values: $b = 0.1$, $k = 8.8$; initial conditions: $X_0 = 0.1$, $Y_0 = 0.1$; Lyapunov exponents: $\lambda_1 \simeq 1.46995$, $\lambda_2 \simeq -3.77254$.
Ref: Schmidt and Wang (1985).

- *Ikeda map :*

$$\begin{cases} X_{n+1} = \gamma + \mu\ (\ X_n\ \cos\phi - Y_n\ \sin\phi\ ) \\ Y_{n+1} = \mu\ (\ X_n\ \sin\phi + Y_n\ \cos\phi\ ) \end{cases} , \tag{19}$$

where $\phi = \beta - \alpha\ /\ (1 + X_n^2 + Y_n^2)$.
Parameter values: $\alpha = 6$, $\beta = 0.4$, $\gamma = 1$, $\mu = 0.9$; initial conditions: $X_0 = 0$, $Y_0 = 0$; Lyapunov exponents: $\lambda_1 \simeq 0.50760$, $\lambda_2 \simeq -0.71832$.
Ref: Ikeda (1979).

- *Sinai map :*

$$\begin{cases} X_{n+1} = X_n + Y_n + \delta\ \cos 2\pi\ Y_n & (\text{ Mod } 1\text{ }) \\ Y_{n+1} = X_n + 2\ Y_n & (\text{ Mod } 1\text{ }) \end{cases} . \tag{20}$$

Parameter value: $\delta = 0.1$; initial conditions: $X_0 = 0.5$, $Y_0 = 0.5$; Lyapunov exponents: $\lambda_1 \simeq 0.95946$, $\lambda_2 \simeq -1.07714$.
Ref: Sinai (1972).

- *Discrete predator-prey map :*

$$\begin{cases} X_{n+1} = X_n\ \exp[\ r\ (\ 1 - X_n/K\ ) - \alpha\ Y_n\ ] & (\text{ Prey }) \\ Y_{n+1} = X_n\ [1 - \exp(-\alpha\ Y_n)\ ] & (\text{ Predator }) \end{cases} . \tag{21}$$

Parameter value: $r = 3$, $K = 1$, $\alpha = 5$; initial conditions: $X_0 = 0.5$, $Y_0 = 0.5$; Lyapunov exponents: $\lambda_1 \simeq 0.19664$, $\lambda_2 \simeq 0.03276$.
Ref: Beddington *et al.* (1975).

1.3 Conservative maps

The conservative maps considered, their parameters, initial condition and the corresponding Lyapunov exponents, are listed below (see Ref. Sprott (2003)). The Fig. 3 show X_n versus Y_n for these two-(three-)dimensional maps.

- *Chirikov standard map :*

$$\begin{cases} X_{n+1} = X_n + Y_{n+1} & (\text{ Mod } 2\pi \text{ }) \\ Y_{n+1} = Y_n + k\ \sin X_n & (\text{ Mod } 2\pi \text{ }) \end{cases} . \tag{22}$$

Parameter value: $k = 1$; initial conditions: $X_0 = 0$, $Y_0 = 6$; Lyapunov exponents: $\lambda_{1,2} \simeq \pm 0.10497$.
Ref: Chirikov (1979).

- *Hénon area–preserving quadratic map :*

$$\begin{cases} X_{n+1} &= X_n \ \cos\alpha - (\ Y_n - X_n^2\ )\ \sin\alpha \\ Y_{n+1} &= X_n \ \sin\alpha + (\ Y_n - X_n^2\ )\ \cos\alpha \end{cases} . \tag{23}$$

Parameter value: $\cos\alpha = 0.24$; Initial conditions: $X_0 = 0.6$, $Y_0 = 0.13$; Lyapunov exponents: $\lambda_{1,2} \simeq \pm 0.00643$.
Ref: Hénon (1969).

- *Arnold's cat map :*

$$\begin{cases} X_{n+1} &= X_n + Y_n \qquad (\text{ Mod } 1\ ) \\ Y_{n+1} &= X_n + k\ Y_n \quad (\text{ Mod } 1\ ) \end{cases} . \tag{24}$$

Parameter value: $k = 2$; initial conditions: $X_0 = 0$, $Y_0 = 1/\sqrt{2}$; Lyapunov exponents: $\lambda_{1,2} = \pm \ln[\frac{1}{2}(3+\sqrt{5})] = \pm 0.96242365\ldots$
Ref: Arnold's and Avez (1968).

- *Gingerbreadman map :*

$$\begin{cases} X_{n+1} &= 1 + \mid X_n \mid - Y_n \\ Y_{n+1} &= X_n \end{cases} . \tag{25}$$

Initial conditions: $X_0 = 0.5$, $Y_0 = 3.7$; Lyapunov exponents: $\lambda_{1,2} \simeq \pm 0.07339$.
Ref: Devaney (1984).

- *Chaotic web map :*

$$\begin{cases} X_{n+1} &= X_n \ \cos\alpha - (\ Y_n + k\ sinX_n\ )\ \sin\alpha \\ Y_{n+1} &= X_n \ \sin\alpha + (\ Y_n + k\ sinX_n\ )\ \cos\alpha \end{cases} . \tag{26}$$

Parameter value: $\alpha = \pi/2$, $k = 1$; initial conditions: $X_0 = 0$, $Y_0 = 3$; Lyapunov exponents: $\lambda_{1,2} \simeq \pm 0.04847$.
Ref: Chernikov *et al.* (1988).

- *Lorenz three–dimensional chaotic map :*

$$\begin{cases} X_{n+1} &= X_n\ Y_n - Z_n \\ Y_{n+1} &= X_n \\ Z_{n+1} &= Y_n \end{cases} . \tag{27}$$

Initial conditions: $X_0 = 0.5$, $Y_0 = 0.5$, $Z_0 = -1$; Lyapunov exponents: $\lambda_{1,2,3} \simeq 0.07456, 0, -0.07456$.
Ref: Lorenz (1993).

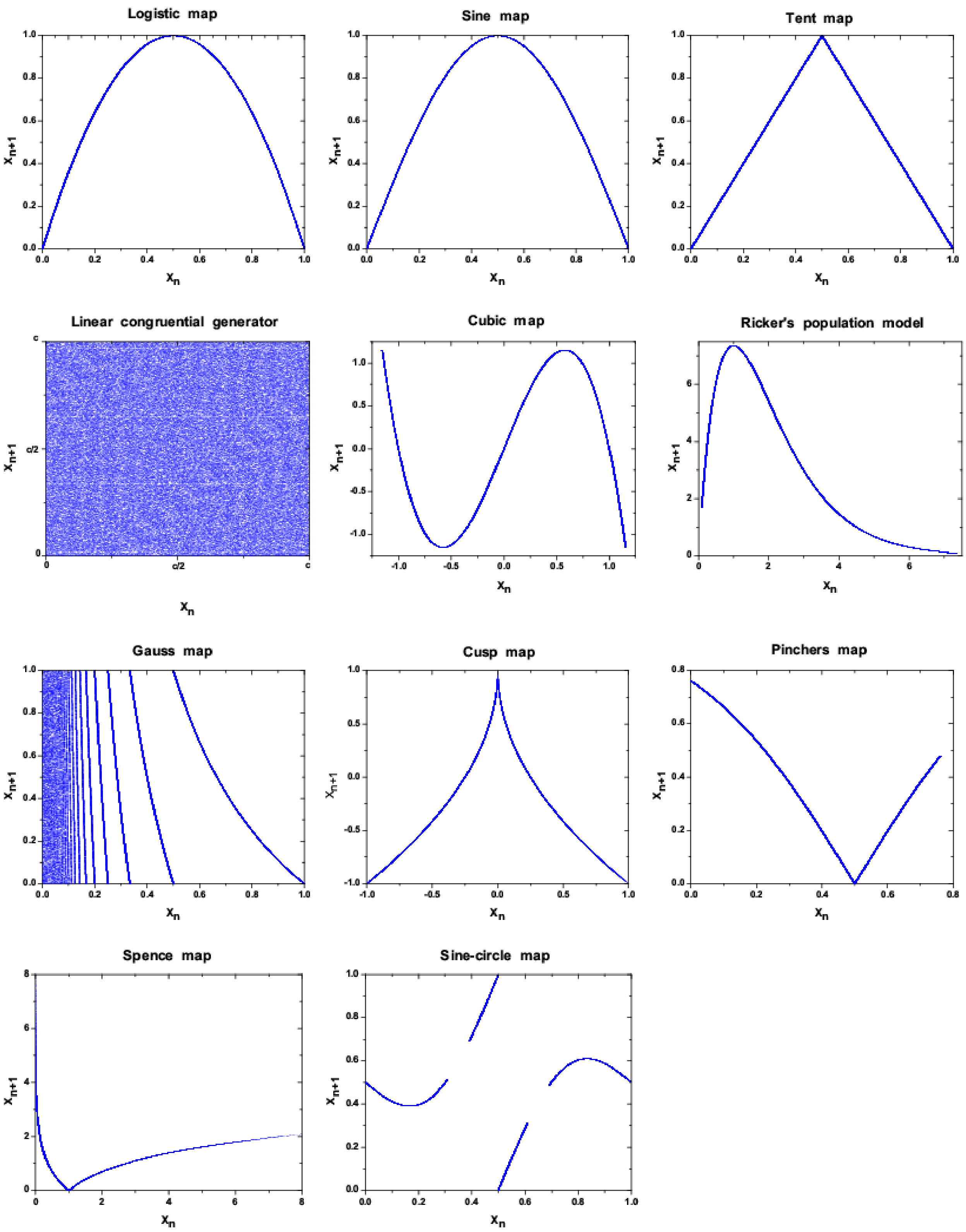

Figure 1. Graphical representation for the eleven noninvertible chaotic maps considered in the present work. The graphs display X_{n+1} versus X_n for the one-dimensional maps (See Sec. 1.1).

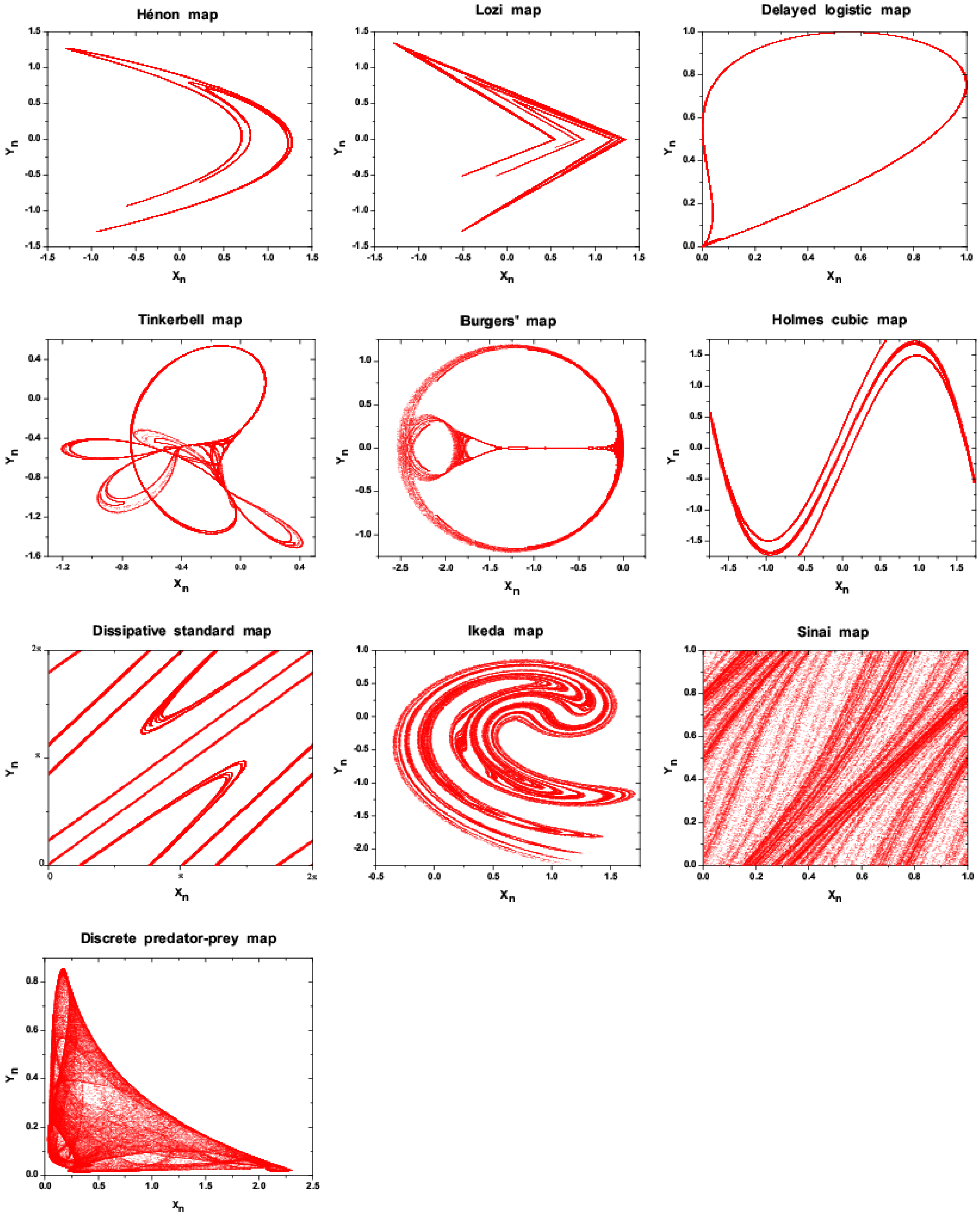

Figure 2. Graphical representation for the ten dissipative chaotic maps considered in the present work. The graphs display Y_n versus X_n for the two-dimensional maps (See Sec. 1.2).

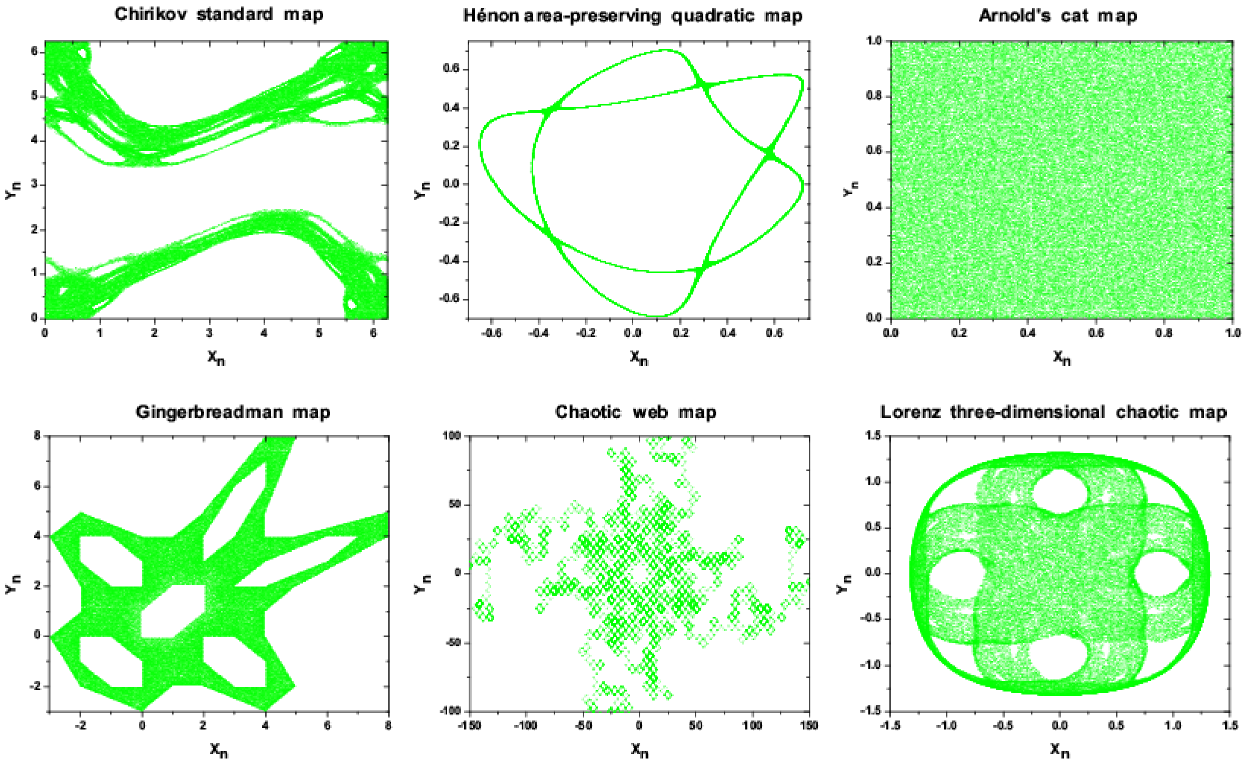

Figure 3. Graphical representation for the six conservative chaotic maps considered in the present work. The graphs display Y_n versus X_n for the two (three)-dimensional maps (See Sec. 1.3).

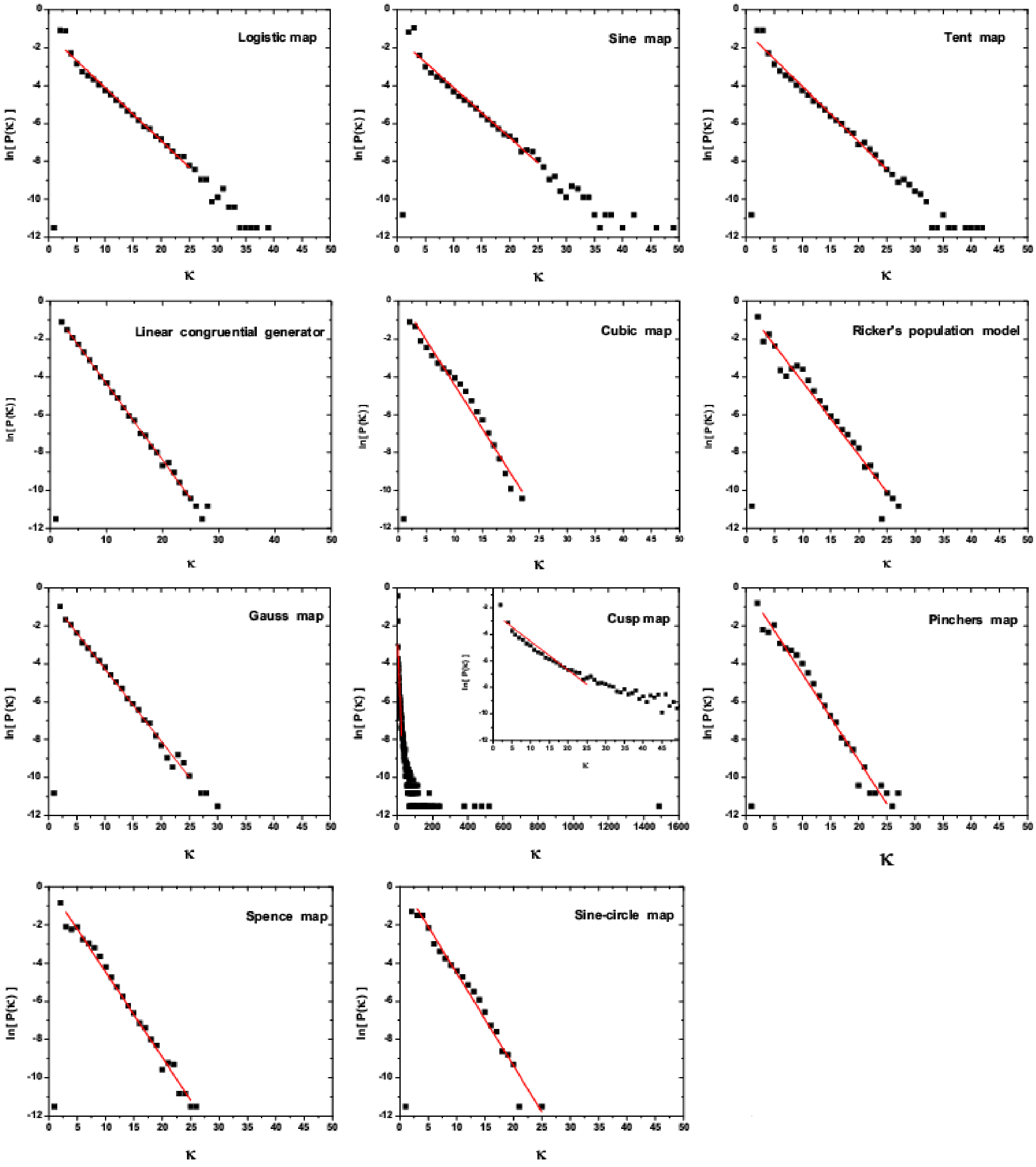

Figure 4. λ-value determination for the noninvertible chaotic maps. In all cases, time series with $N = 10^5$ are considered, and linear scaling regions are defined by $3 \leq \kappa \leq 25$ or $3 \leq \kappa \leq \kappa_{min}$ if $\kappa_{mim} < 25$.

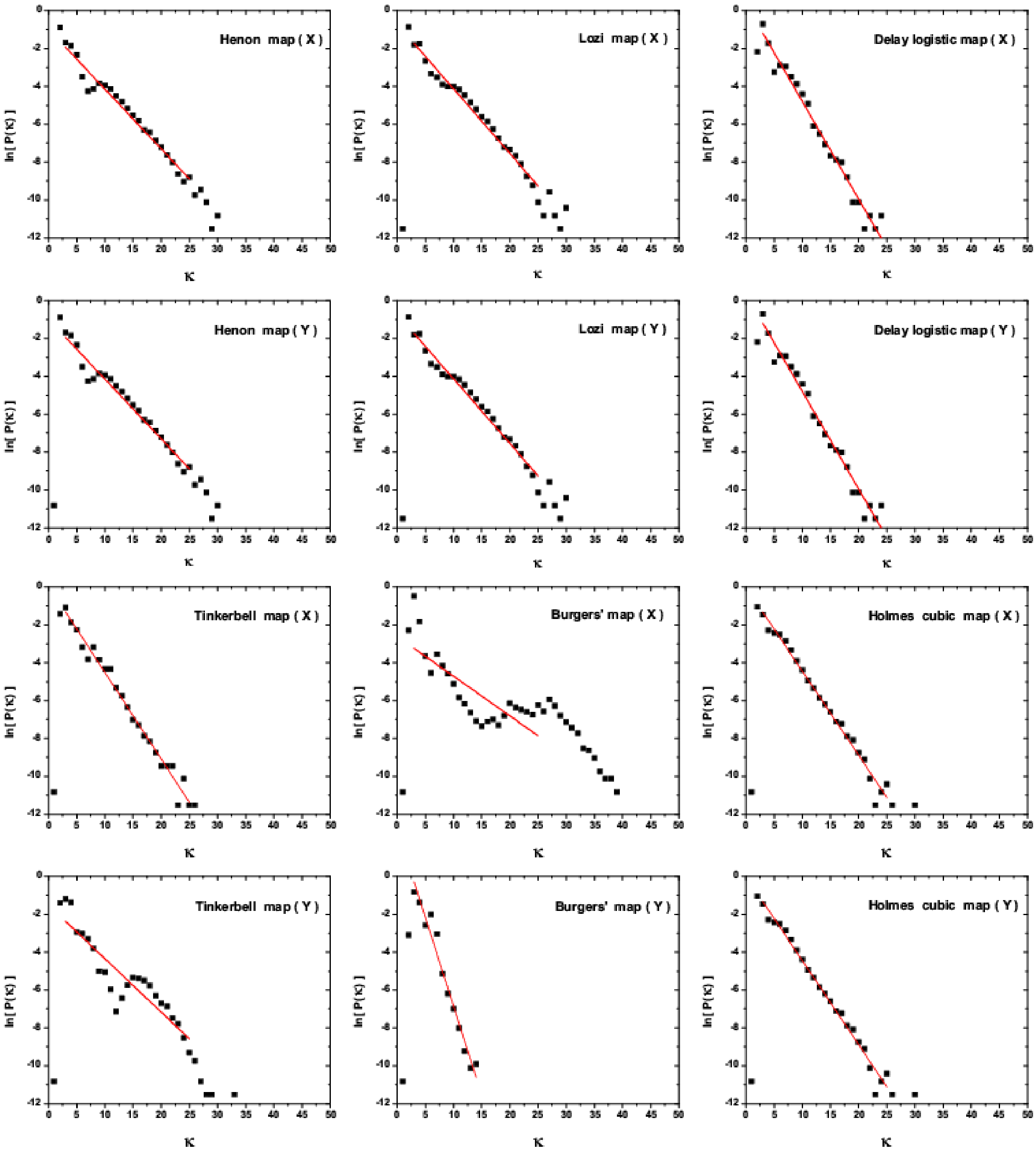

Figure 5. λ-value determination for the dissipative chaotic maps. In all cases, time series with $N = 10^5$ are considered, and linear scaling regions are defined by $3 \leq \kappa \leq 25$ or $3 \leq \kappa \leq \kappa_{min}$ if $\kappa_{mim} < 25$. (Continued on next page.)

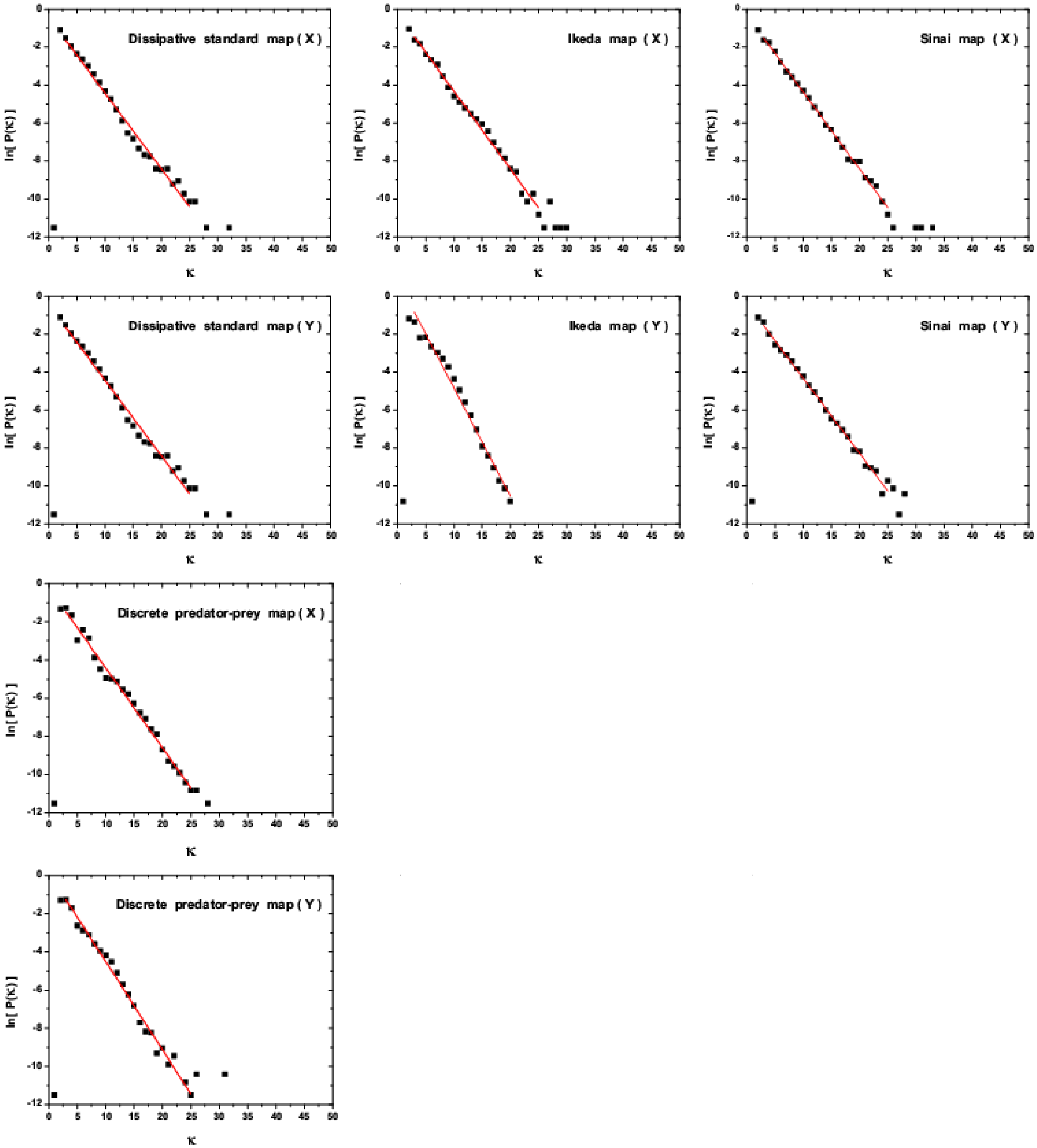

Figure 5. λ-value determination for the dissipative chaotic maps. In all cases, time series with $N = 10^5$ are considered, and linear scaling regions are defined by $3 \leq \kappa \leq 25$ or $3 \leq \kappa \leq \kappa_{min}$ if $\kappa_{mim} < 25$. (Continued from previous page.)

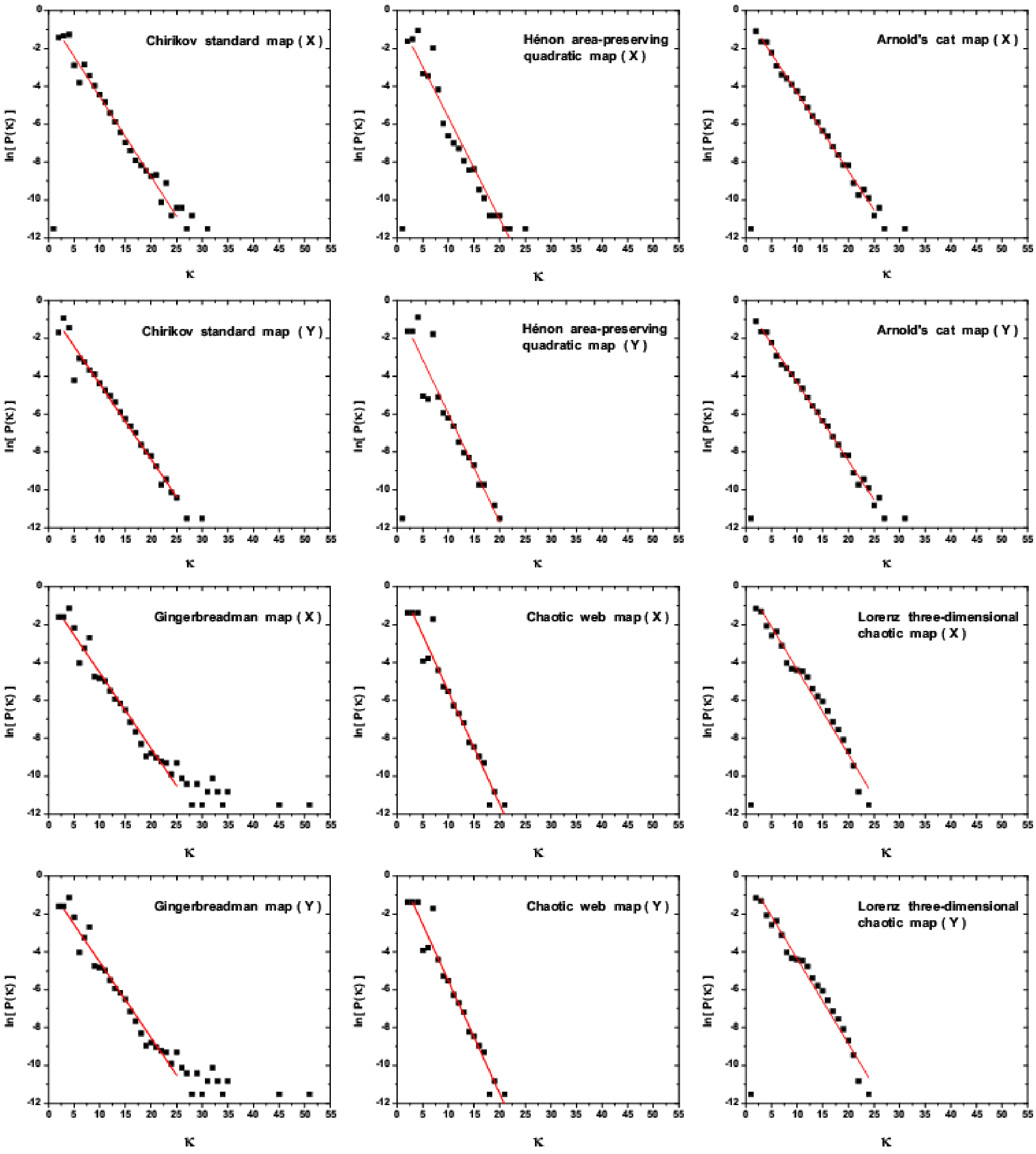

Figure 6. λ-value determination for the conservative chaotic maps. In all cases, time series with $N = 10^5$ are considered, and linear scaling regions are defined by $3 \leq \kappa \leq 25$ or $3 \leq \kappa \leq \kappa_{min}$ if $\kappa_{mim} < 25$. (Continued on next page.)

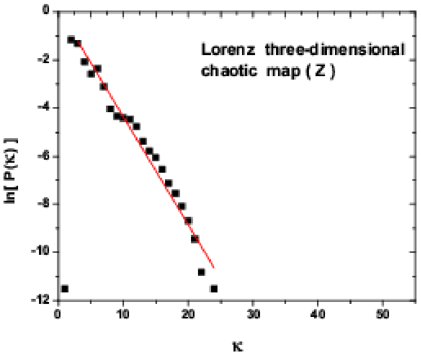

Figure 6. λ-value determination for the conservative chaotic maps. In all cases, time series with $N = 10^5$ are considered, and linear scaling regions are defined by $3 \leq \kappa \leq 25$ or $3 \leq \kappa \leq \kappa_{min}$ if $\kappa_{mim} < 25$. (Continued from previous page.)

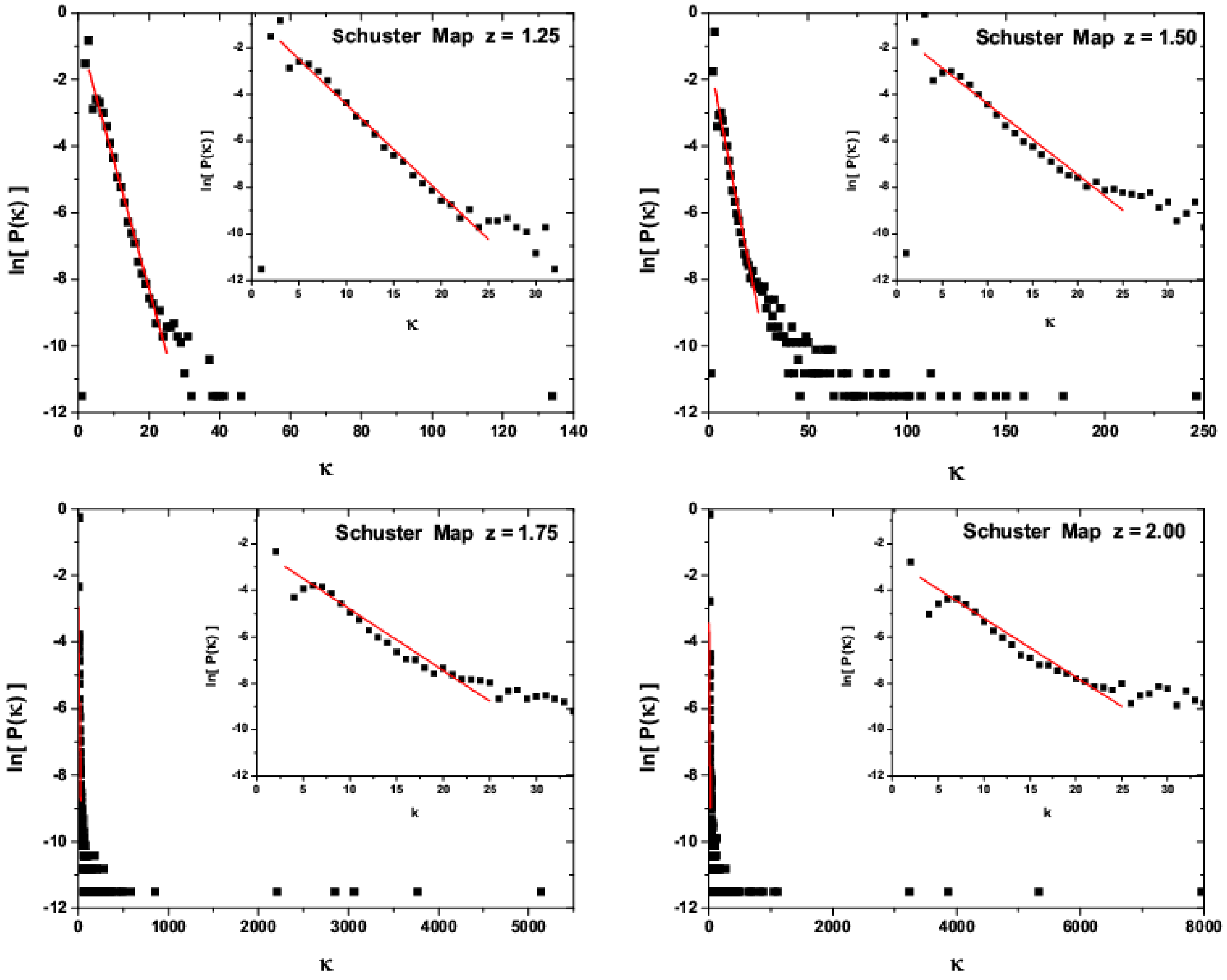

Figure 7. λ-value determination for the Schuster maps. In all cases, time series with $N = 10^5$ are considered, and linear scaling regions are defined by $3 \leq \kappa \leq 25$ or $3 \leq \kappa \leq \kappa_{min}$ if $\kappa_{mim} < 25$.

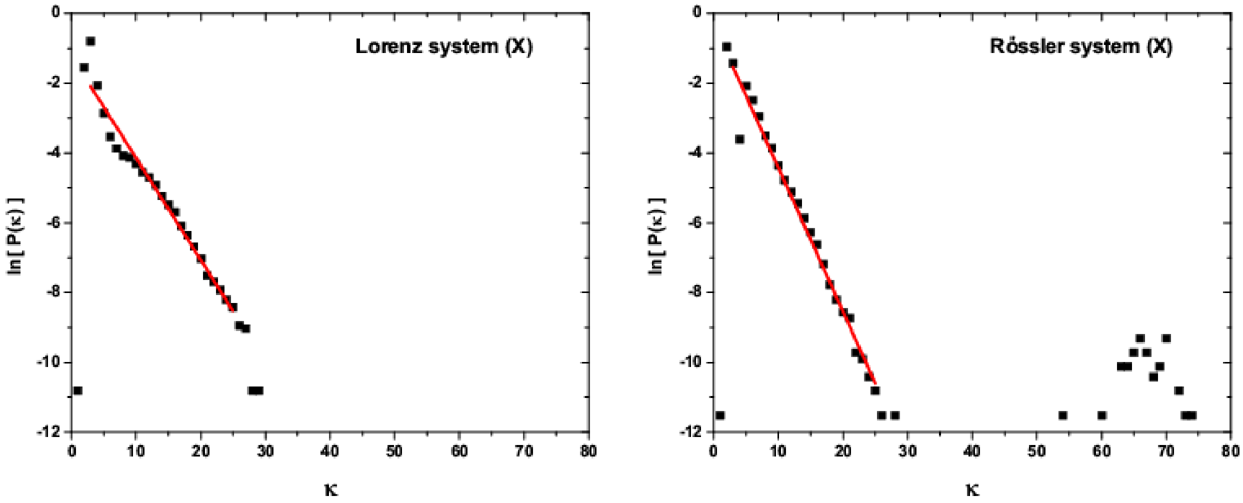

Figure 8. λ-value determination for the chaotic flows (Lorenz and Rössler systems). In all cases, time series with $N = 10^5$ are considered, and linear scaling regions are defined by $3 \leq \kappa \leq 25$.

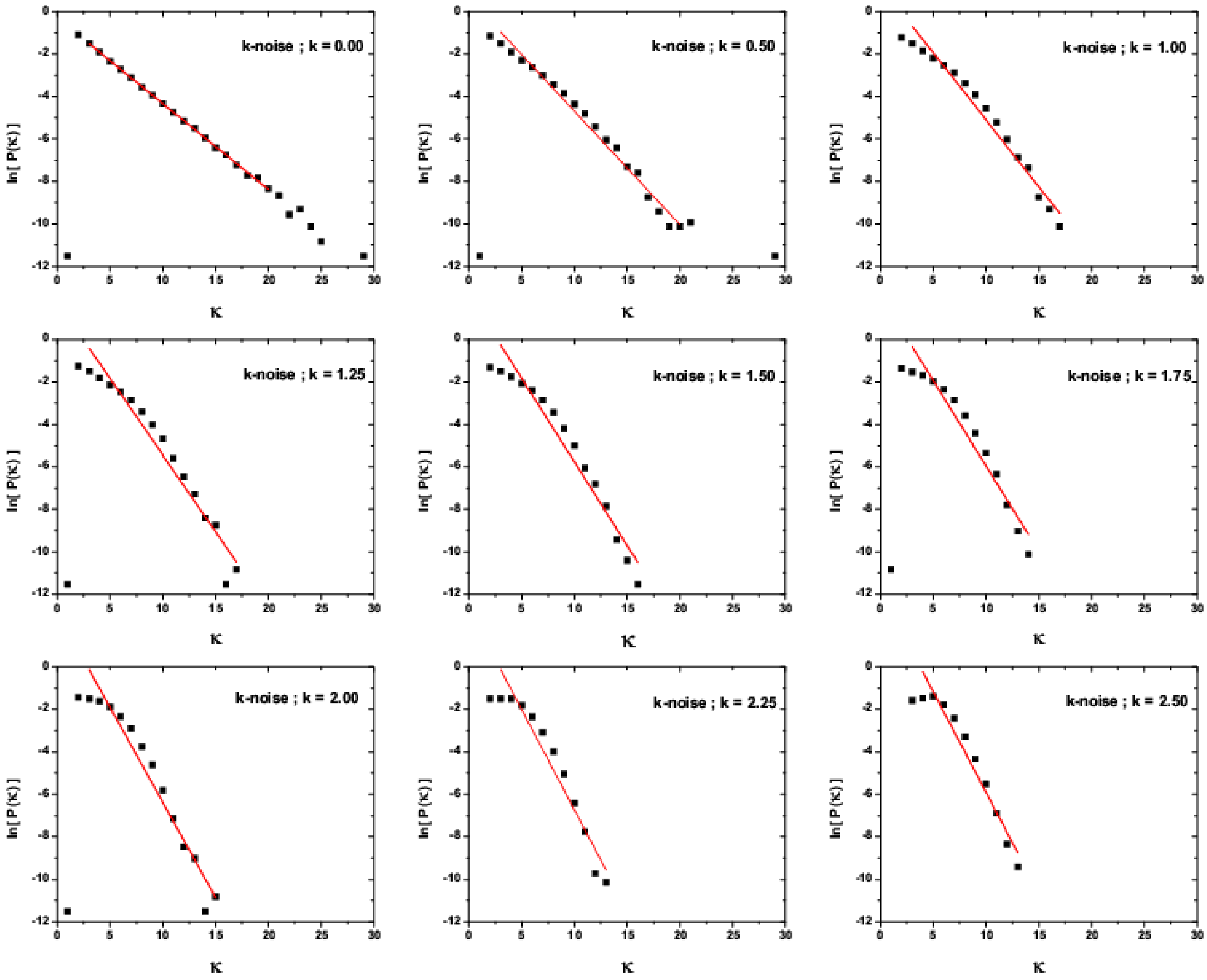

Figure 9. λ-value determination for the k-noise. In all cases, time series with $N = 10^5$ are considered, and linear scaling regions are defined by $3 \leq \kappa \leq 20$ or $3 \leq \kappa \leq \kappa_{min}$ if $\kappa_{mim} < 20$.

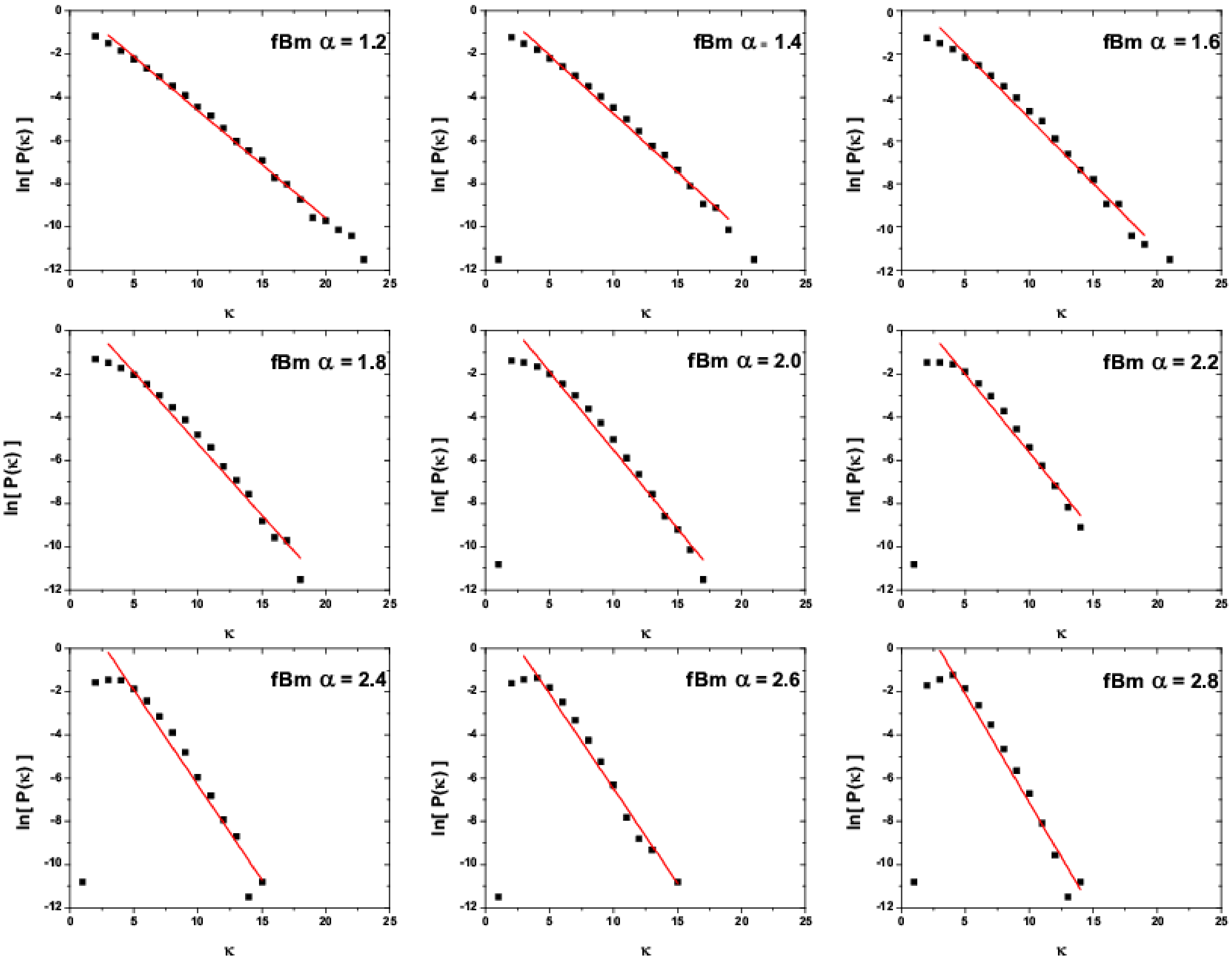

Figure 10. λ-value determination for the fBm noise. In all cases, time series with $N = 10^5$ are considered, and linear scaling regions are defined by $3 \leq \kappa \leq 20$ or $3 \leq \kappa \leq \kappa_{min}$ if $\kappa_{mim} < 20$.

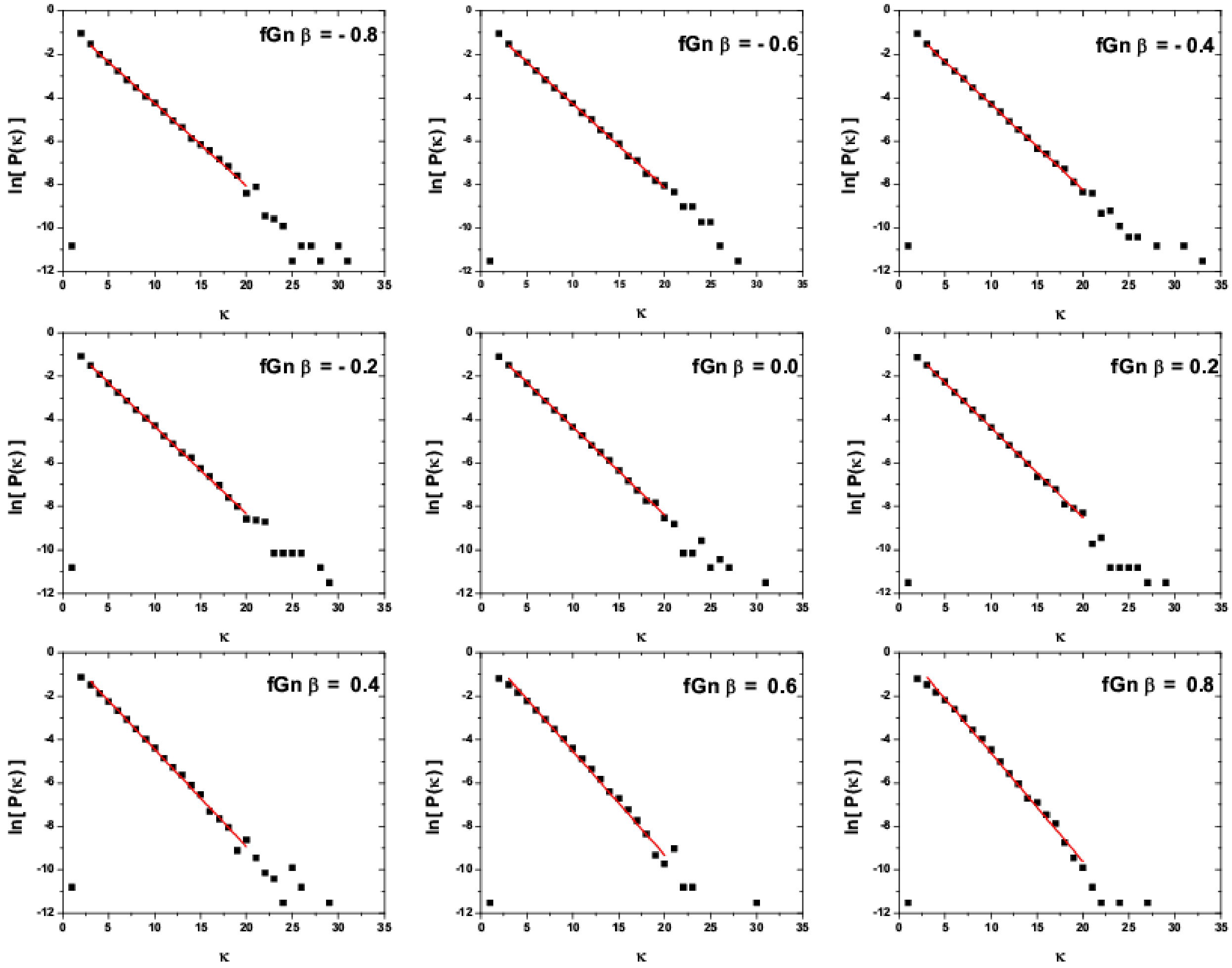

Figure 11. λ-value determination for the fGn noise. In all cases, time series with $N = 10^5$ are considered, and linear scaling regions are defined by $3 \leq \kappa \leq 20$ or $3 \leq \kappa \leq \kappa_{min}$ if $\kappa_{mim} < 20$.

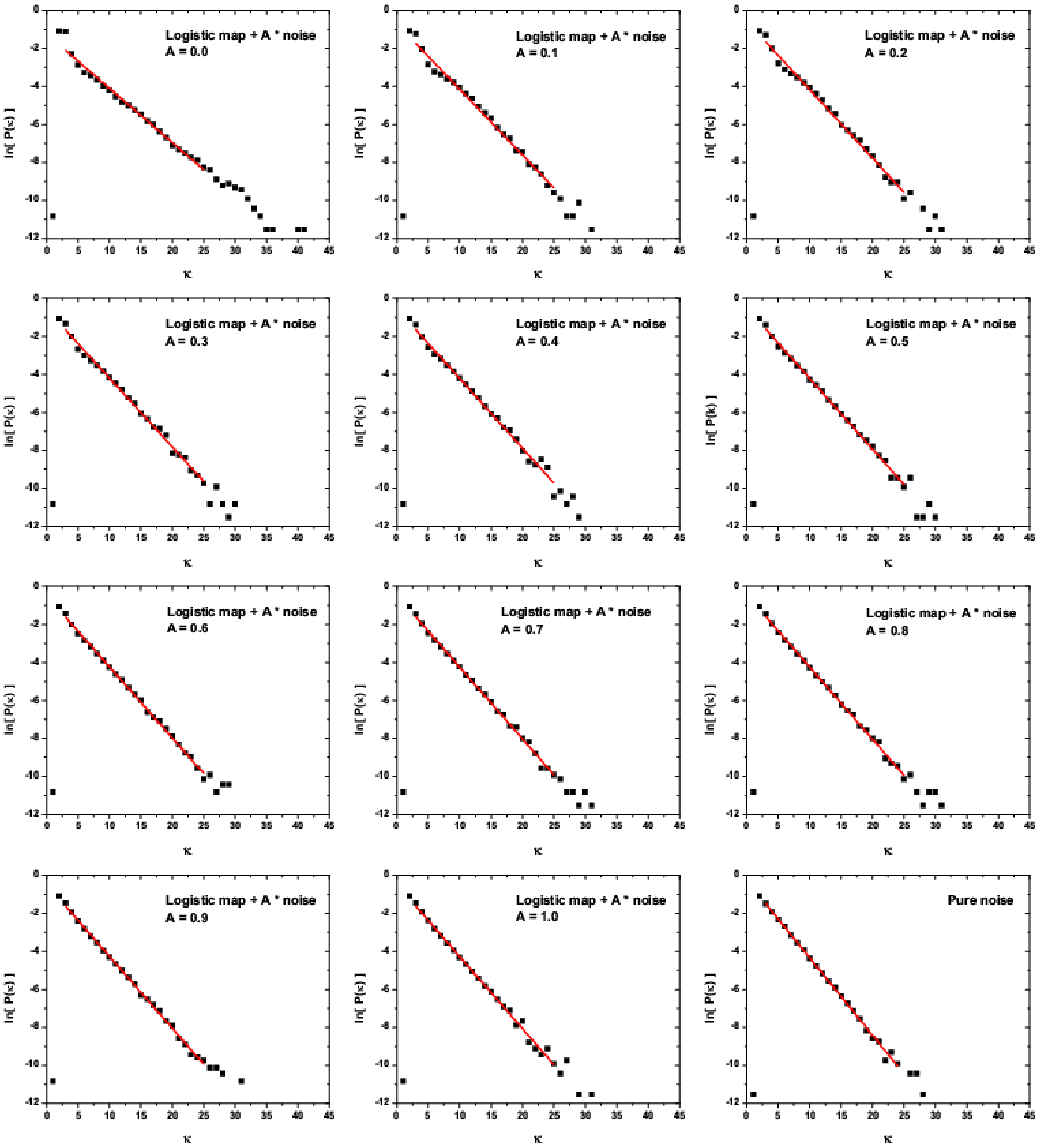

Figure 12. λ-value determination for the logistic map ($\rho = 4$) contaminated with additive noise (uncorrelated flat PDF) of amplitude $0 \leq A \leq 1$ with step $\Delta A = 0.1$. The corresponding determination for pure noise is also included in the figure. In all cases, time series with $N = 10^5$ are considered, and linear scaling regions are defined by $3 \leq \kappa \leq 25$.

Table 1. Obtained quantifier values for all systems studied: node cut-off k_{max}; skewness $\widehat{\gamma_1}(\xi)$; kurtosis $\widehat{\gamma_2}(\xi, \varrho)$; λ parameter; LCL: lower confidence interval value for parameter λ; UCL: upper confidence interval value for parameter λ; goodness of the fit of λ parameter R^2 ; Shannon entropy value $\mathcal{S}[P]$; Fisher information value $\mathcal{F}[P]$; normalized Shannon entropy value using white noise $\mathcal{S}/\mathcal{S}_{wn}$; normalized Shannon entropy value using white gaussian noise $\mathcal{S}/\mathcal{S}_{wgn}$.

**Systems**	k_{max}	**skewness**	**kurtosis**	λ	**LCL**	**UCL**	R^2	$\mathcal{S}[P]$	$\mathcal{F}[P]$	$\mathcal{S}/\mathcal{S}_{wn}$	$\mathcal{S}/\mathcal{S}_{wgn}$
**Noninvertible maps**											
Logistic map	39	0.666	2.333	0.281	0.264	0.298	0.982	1.847	0.204	0.968	0.967
Sine map	49	0.666	2.500	0.265	0.244	0.287	0.968	1.805	0.210	0.945	0.945
Tent map	42	0.666	2.333	0.285	0.268	0.302	0.982	1.846	0.203	0.967	0.967
Lineal congruential generator	28	0.600	2.200	0.405	0.397	0.413	0.998	1.909	0.182	1.000	1.000
Cubic map	22	0.666	1.666	0.469	0.431	0.507	0.974	1.899	0.185	0.995	0.994
Ricker's population model	27	0.714	1.714	0.387	0.350	0.425	0.955	1.824	0.292	0.955	0.955
Gauss map	30	0.600	2.200	0.382	0.365	0.398	0.992	1.902	0.212	0.996	0.996
Cusp map	1487	0.500	5.250	0.217	0.175	0.259	0.839	1.343	0.348	0.704	0.704
Pincher's map	27	0.666	1.667	0.457	0.423	0.490	0.973	1.860	0.251	0.974	0.974
Spence map	26	0.666	1.666	0.448	0.426	0.470	0.988	1.859	0.266	0.974	0.974
Sine-circle map	25	0.600	2.000	0.484	0.445	0.522	0.974	1.880	0.156	0.985	0.985
**Dissipative Maps**											
Hénon map (X)	30	0.666	2.166	0.316	0.291	0.341	0.969	1.854	0.242	0.971	0.971
Hénon map (Y)	30	0.666	2.166	0.316	0.291	0.341	0.969	1.854	0.239	0.971	0.971
Lozi map (X)	30	0.666	2.166	0.341	0.318	0.364	0.978	1.860	0.251	0.974	0.974
Lozi map (Y)	30	0.666	2.166	0.341	0.318	0.364	0.978	1.860	0.251	0.974	0.974
Delay logistic map (X)	24	0.600	1.800	0.515	0.478	0.551	0.976	1.659	0.191	0.869	0.869
Delay logistic map (Y)	24	0.600	1.800	0.515	0.478	0.551	0.976	1.659	0.191	0.869	0.869
Tinkerbell map (X)	26	0.666	1.666	0.457	0.429	0.485	0.982	1.851	0.156	0.970	0.969
Tinkerbell map (Y)	33	0.600	3.000	0.282	0.224	0.340	0.823	1.805	0.164	0.945	0.945
Burgers' map (X)	39	0.500	5.750	0.209	0.134	0.285	0.594	1.415	0.264	0.741	0.741
Burgers' map (Y)	14	0.500	1.250	0.936	0.822	1.051	0.968	1.513	0.186	0.792	0.792
Holmes map (X)	30	0.600	2.000	0.444	0.418	0.469	0.984	1.895	0.196	0.993	0.993
Holmes map (Y)	30	0.600	2.000	0.444	0.418	0.469	0.984	1.895	0.196	0.993	0.993
Dissipative standard map (X)	29	0.600	2.200	0.392	0.383	0.401	0.997	1.908	0.190	0.999	0.999
Dissipative standard map (Y)	32	0.600	2.000	0.399	0.378	0.419	0.987	1.908	0.183	0.999	0.999
Ikeda map (X)	30	0.600	2.200	0.409	0.393	0.426	0.992	1.905	0.195	0.998	0.998
Ikeda map (Y)	20	0.600	1.800	0.570	0.533	0.607	0.984	1.893	0.173	0.992	0.992
Sinai map (X)	33	0.600	2.200	0.406	0.395	0.418	0.996	1.905	0.187	0.998	0.998
Sinai map (Y)	28	0.600	2.200	0.396	0.382	0.410	0.994	1.903	0.180	0.997	0.996

Continued on next page

Table 1 – *Continued from previous page*

**Systems**	k_{max}	**skewness**	**kurtosis**	λ	**LCL**	**UCL**	R^2	$\mathcal{S}[P]$	$\mathcal{F}[P]$	$\mathcal{S}/\mathcal{S}_{wn}$	$\mathcal{S}/\mathcal{S}_{wgn}$
Discrete predator-prey map (X)	28	0.600	2.200	0.419	0.400	0.439	0.989	1.863	0.167	0.976	0.976
Discrete predator-prey map (Y)	31	0.600	2.000	0.463	0.441	0.484	0.989	1.886	0.157	0.988	0.988
**Conservative Maps**											
Chirikov standard map (X)	31	0.600	2.000	0.421	0.392	0.451	0.976	1.812	0.173	0.949	0.949
Chirikov standard map (Y)	30	0.600	2.200	0.397	0.366	0.427	0.971	1.734	0.194	0.908	0.908
Hnon-area preserving quadratic map (X)	25	0.200	1.200	0.533	0.465	0.602	0.929	1.633	0.240	0.855	0.855
Hnon-area preserving quadratic map (Y)	20	0.200	1.200	0.571	0.466	0.676	0.893	1.449	0.385	0.759	0.759
Arnold's cat map (X)	31	0.200	2.200	0.411	0.399	0.424	0.995	1.899	0.187	0.995	0.995
Arnold's cat map (Y)	31	0.200	2.200	0.411	0.399	0.424	0.995	1.899	0.187	0.995	0.995
Gingerbreadman map (X)	51	0.333	1.666	0.397	0.360	0.435	0.957	1.832	0.174	0.959	0.959
Gingerbreadman map (Y)	51	0.333	1.666	0.397	0.360	0.435	0.957	1.832	0.174	0.959	0.959
Chaotic web map (X)	21	0.600	1.400	0.594	0.523	0.665	0.949	1.647	0.278	0.863	0.863
Chaotic web map (Y)	21	0.600	1.400	0.594	0.523	0.665	0.949	1.647	0.278	0.863	0.863
Lorenz three dimensional chaotic map (X)	24	0.600	2.200	0.448	0.412	0.485	0.971	1.888	0.182	0.989	0.989
Lorenz three dimensional chaotic map (Y)	24	0.600	2.200	0.448	0.412	0.485	0.971	1.888	0.182	0.989	0.989
Lorenz three dimensional chaotic map (Z)	24	0.600	2.200	0.448	0.412	0.485	0.971	1.888	0.182	0.989	0.989
**Schuster Maps**											
z = 1.25	134	0.600	2.000	0.387	0.362	0.412	0.979	1.762	0.223	0.923	0.923
z = 1.50	246	0.600	2.600	0.306	0.271	0.340	0.938	1.569	0.308	0.822	0.822
z = 1.75	5139	0.500	3.500	0.263	0.215	0.312	0.853	1.049	0.499	0.549	0.549
z = 2.00	7960	-1.000	13.000	0.252	0.196	0.308	0.797	0.751	0.617	0.394	0.394
**Chaotic Flows**											
Lorenz system (X)	29	0.600	2.800	0.292	0.267	0.317	0.964	1.745	0.189	0.914	0.914
Rössler system (X)	74	0.333	1.833	0.412	0.385	0.439	0.979	1.826	0.274	0.956	0.956
**Noise** f^{-k}											
k = 0.00	29	0.600	2.200	0.404	0.398	0.409	0.999	1.909	0.181	1.000	1.000
k = 0.50	29	0.600	1.800	0.534	0.499	0.569	0.984	1.905	0.171	0.998	0.998
k = 1.00	17	0.600	1.600	0.628	0.565	0.692	0.970	1.894	0.162	0.992	0.992
k = 1.25	17	0.600	1.600	0.719	0.623	0.816	0.949	1.885	0.154	0.987	0.987
k = 1.50	16	0.200	1.600	0.787	0.679	0.896	0.951	1.871	0.148	0.980	0.980
k = 1.75	14	0.200	1.400	0.804	0.666	0.941	0.939	1.852	0.138	0.970	0.970
k = 2.00	15	0.000	1.750	0.895	0.761	1.029	0.947	1.830	0.131	0.959	0.959
k = 2.25	13	0.000	1.500	0.945	0.767	1.122	0.935	1.787	0.128	0.936	0.936
k = 2.50	12	0.000	1.500	0.946	0.766	1.126	0.942	1.739	0.125	0.911	0.911

Continued on next page

Table 1 – *Continued from previous page*

**Systems**	k_{max}	**skewness**	**kurtosis**	λ	**LCL**	**UCL**	R^2	$\mathcal{S}[P]$	$\mathcal{F}[P]$	$\mathcal{S}/\mathcal{S}_{wn}$	$\mathcal{S}/\mathcal{S}_{wgn}$
**Noise fBm**											
$\alpha = 1.2$	23	0.600	2.000	0.499	0.478	0.520	0.983	1.905	0.170	0.998	0.998
$\alpha = 1.4$	21	0.600	1.800	0.541	0.511	0.572	0.989	1.899	0.162	0.995	0.995
$\alpha = 1.6$	21	0.600	1.800	0.602	0.561	0.642	0.984	1.891	0.156	0.990	0.990
$\alpha = 1.8$	18	0.200	1.600	0.662	0.605	0.719	0.977	1.880	0.148	0.985	0.985
$\alpha = 2.0$	17	0.200	1.600	0.726	0.660	0.792	0.976	1.860	0.136	0.974	0.974
$\alpha = 2.2$	14	0.000	1.750	0.724	0.640	0.809	0.971	1.833	0.128	0.960	0.960
$\alpha = 2.4$	15	0.000	1.750	0.877	0.754	1.001	0.953	1.794	0.121	0.940	0.940
$\alpha = 2.6$	15	0.000	1.500	0.877	0.783	0.972	0.975	1.742	0.120	0.913	0.913
$\alpha = 2.8$	14	0.000	1.500	1.008	0.873	1.143	0.962	1.681	0.121	0.880	0.880
**Noise fGn**											
$\beta = 0.8$	31	0.600	2.200	0.380	0.370	0.390	0.997	1.907	0.195	0.999	0.999
$\beta = 0.6$	28	0.600	2.200	0.386	0.380	0.392	0.999	1.908	0.193	0.999	0.999
$\beta = 0.4$	33	0.600	2.200	0.392	0.386	0.398	0.999	1.909	0.189	1.000	1.000
$\beta = 0.2$	29	0.600	2.200	0.402	0.393	0.410	0.998	1.909	0.185	1.000	1.000
$\beta = 0.0$	31	0.600	2.200	0.407	0.400	0.414	0.999	1.909	0.183	1.000	1.000
$\beta = -0.2$	29	0.600	2.200	0.414	0.405	0.423	0.999	1.909	0.177	1.000	1.000
$\beta = -0.4$	29	0.600	2.000	0.448	0.428	0.467	0.993	1.908	0.172	0.999	0.999
$\beta = -0.6$	30	0.600	2.000	0.479	0.459	0.499	0.993	1.906	0.166	0.998	0.998
$\beta = -0.8$	27	0.600	1.800	0.499	0.480	0.517	0.995	1.902	0.160	0.996	0.996
**Noise Contamination:** $Logistic\ map\ +\ A\ \times\ white - noise$											
$A = 0.0$	41	0.666	2.333	0.285	0.268	0.302	0.982	1.846	0.203	0.967	0.967
$A = 0.1$	34	0.666	2.167	0.345	0.331	0.360	0.991	1.876	0.195	0.967	0.982
$A = 0.2$	31	0.666	2.000	0.360	0.345	0.374	0.992	1.886	0.193	0.988	0.988
$A = 0.3$	30	0.666	2.000	0.362	0.351	0.373	0.995	1.895	0.191	0.993	0.993
$A = 0.4$	29	0.666	2.000	0.367	0.350	0.383	0.990	1.900	0.190	0.995	0.995
$A = 0.5$	30	0.333	2.000	0.371	0.363	0.380	0.997	1.903	0.189	0.997	0.997
$A = 0.6$	29	0.333	2.000	0.374	0.366	0.382	0.998	1.905	0.188	0.998	0.998
$A = 0.7$	31	0.333	2.000	0.378	0.369	0.387	0.997	1.906	0.187	0.998	0.998
$A = 0.8$	31	0.333	2.000	0.379	0.371	0.387	0.998	1.907	0.186	0.999	0.999
$A = 0.9$	31	0.333	1.833	0.378	0.371	0.386	0.998	1.908	0.185	0.999	0.999
$A = 1.0$	31	0.333	1.833	0.379	0.366	0.391	0.994	1.908	0.184	0.999	0.999
Pure noise	36	0.333	1.833	0.407	0.396	0.417	0.997	1.909	0.181	1.000	1.000
